# Lagged Coupled Changes Between White Matter Microstructure and Processing Speed in Healthy Aging: A Longitudinal Investigation

**DOI:** 10.3389/fnagi.2019.00298

**Published:** 2019-11-21

**Authors:** Jessica Oschwald, Susan Mérillat, Franziskus Liem, Christina Röcke, Mike Martin, Lutz Jäncke

**Affiliations:** ^1^University Research Priority Program “Dynamics of Healthy Aging”, University of Zurich, Zurich, Switzerland; ^2^Division of Gerontopsychology, Psychological Institute, University of Zurich, Zurich, Switzerland; ^3^Division of Neuropsychology, Psychological Institute, University of Zurich, Zurich, Switzerland

**Keywords:** white matter microstructure, processing speed, longitudinal, coupled changes, healthy aging, fractional anisotropy, latent change score model, structural equation modeling (SEM)

## Abstract

Age-related differences in white matter (WM) microstructure have been linked to lower performance in tasks of processing speed in healthy older individuals. However, only few studies have examined this link in a longitudinal setting. These investigations have been limited to the correlation of simultaneous changes in WM microstructure and processing speed. Still little is known about the nature of age-related changes in WM microstructure, i.e., regionally distinct vs. global changes. In the present study, we addressed these open questions by exploring whether previous changes in WM microstructure were related to subsequent changes in processing speed: (a) 1 year later; or (b) 2 years later. Furthermore, we investigated whether age-related changes in WM microstructure were regionally specific or global. We used data from four occasions (covering 4 years) of the Longitudinal Healthy Aging Brain (LHAB) database project (*N* = 232; age range at baseline = 64–86). As a measure of WM microstructure, we used mean fractional anisotropy (FA) in 10 major WM tracts averaged across hemispheres. Processing speed was measured with four cognitive tasks. Statistical analyses were conducted with bivariate latent change score (LCS) models. We found, for the first time, evidence for lagged couplings between preceding changes in FA and subsequent changes in processing speed 2 years, but not 1 year later in some of the WM tracts (anterior thalamic radiation, superior longitudinal fasciculus). Our results supported the notion that FA changes were different between regional WM tracts rather than globally shared, with some tracts showing mean declines in FA, and others remaining relatively stable across 4 years.

## Introduction

It is widely recognized that fluid cognitive abilities decline during the course of aging (Park et al., 2002; Deary et al., 2009; Salthouse, 2010), with substantial variability observed between individuals (e.g., Wilson et al., 2002). Processing speed, or the speeded performance in simple cognitive or motor tasks, is a fluid cognitive ability which is particularly vulnerable to the effects of age (e.g., Schaie, 2005). Processing speed has been found to explain a substantial proportion of the performance variability in higher-order cognitive abilities, such as working memory, or executive functioning (Schaie, 1989; Salthouse, 1996; Verhaeghen and Salthouse, 1997; Zimprich and Martin, 2002). The investigation of the neural mechanisms underlying changes in processing speed is therefore highly relevant in order to advance our understanding of age-related decline in fluid cognitive abilities in general.

In previous research, age-related cortical disconnection caused by the degradation of the microstructure of myelinated axonal fiber bundles that make up the white matter (WM) of the brain has been identified as one potential neural mechanism for age-related deficits in processing speed (Madden et al., 2009, 2012; Bennett and Madden, 2014). The microstructural properties of these WM fiber pathways can be estimated *in vivo* with diffusion-weighted magnetic resonance imaging (DW-MRI), a neuroimaging method that allows to measure the rate and directionality of water diffusion in brain tissues (Jones et al., 2013). A measure commonly derived from DW-MRI is fractional anisotropy (FA) that captures the directionality of diffusion within a tissue independent of the rate of diffusion (Rosenbloom et al., 2003). FA has proven to be a sensitive measure of aging, with average declines observed after 1 year in healthy older adults (Teipel et al., 2010). Several cross-sectional studies have reported that age-related deficits in processing speed are associated with impaired WM microstructure across widespread regions of the brain (Vernooij et al., 2009; Penke et al., 2010, 2012; Kerchner et al., 2012; Haász et al., 2013; Kuznetsova et al., 2016; Hirsiger et al., 2017), implying that a global degradation of WM microstructure is related to cognitive slowing in old age (but see: Bucur et al., 2008; Kennedy and Raz, 2009; Salami et al., 2012). However, cross-sectional studies are not sufficient to establish an association between age-related changes in two developmental variables since they might only be correlated due to a common mean trend, such as decline, over time (Hofer et al., 2006; Lindenberger et al., 2011). Longitudinal studies and appropriate statistical methods to estimate inter-individual differences in intra-individual change are necessary to disentangle developmental change relations (Oschwald et al., 2019). A method well-suited for this purpose is latent change score (LCS) modeling implemented in the structural equation modeling (SEM) framework. In brief, LCS models estimate LCS between subsequent measurement scores that represent true change separated from measurement error (McArdle, 2009). Furthermore, these models allow the estimation of dynamic within-person associations between two change processes.

Only few longitudinal studies have directly associated age-related changes in WM microstructure to changes in processing speed (Charlton et al., 2010; Lövdén et al., 2014; Ritchie et al., 2015; Gorbach et al., 2017; Song et al., 2018). Of these, only two have used LCS modeling to estimate inter-individual differences in intra-individual change (Lövdén et al., 2014; Ritchie et al., 2015). Lövdén et al. (2014) reported significant positive change-change associations between processing speed and WM microstructure in the corticospinal tract, such that individuals with higher FA declines in this tract also showed steeper declines in processing speed across 2 years. In contrast, Ritchie et al. (2015) found only positive level-change relationships between global FA and subsequent 3-year changes in processing speed, but no change-change relationships between these measures. Considering these two studies it becomes obvious that the limited longitudinal evidence arrives at very different conclusions regarding the FA-processing speed association. It is important to note that both studies included only two measurement occasions. Thus, they were limited to the investigation of simultaneous relations between differences in FA and processing speed. However, based on prominent theories of cognitive aging, one may expect that healthy older individuals are able—at least in part—to compensate for age-related degradation of brain structure and function, e.g., by recruiting secondary neural networks or by adopting certain cognitive strategies (Stern, 2002, 2009; Park and Reuter-Lorenz, 2009; Reuter-Lorenz and Park, 2014). Thus, we expect that—in healthy older adults—declines in FA lead to observable decrements in processing speed only after a longer time lag, and when compensatory functions are no longer sufficient to counteract brain structural decline.

A satellite topic that has been discussed in the pertinent literature relates to the uniformity of FA and other measures of WM microstructure and its aging-related decline across the brain. Empirical findings have demonstrated that, among older adults, WM microstructure is highly correlated between WM tracts sampled across the entire brain (e.g., Penke et al., 2010, 2012; Cox et al., 2016). This has led researchers to believe that the effects of age on the microstructure of WM might be a relatively global phenomenon, rather than specific to individual WM tracts (Bennett and Madden, 2014). However, several authors have also reported stronger correlations on the level of regional WM tracts (Wahl et al., 2010; Li et al., 2012; Lövdén et al., 2013). Furthermore, only few studies have investigated the uniformity of age-related changes in WM microstructure in a longitudinal setting, with heterogeneous findings, supporting either more regionally (Lövdén et al., 2014), or globally shared changes (Ritchie et al., 2015; Bender et al., 2016).

For the present study, we are using a longitudinal dataset that comprises four occasions of cognitive testing and brain imaging in healthy older adults. With this dataset, we intend to assess dynamic change-change relations between FA and processing speed over a period of 4 years, using LCS modeling. Specifically, we investigate lagged relationships between preceding changes in FA and subsequent changes in processing speed: (a) 1 year; or (b) 2 years later. Based on the assumption that the compensatory mechanisms weaken with time, we expect that the associations will increase with the size of the time lag. In addition, due to previous suggestions that FA in brain-wide WM tracts is often inter-correlated, we will explore whether a global FA factor can capture this shared variance longitudinally, or whether regional differences in FA changes outweigh shared effects of aging.

## Materials and Methods

### Participants

**Figure 1:**
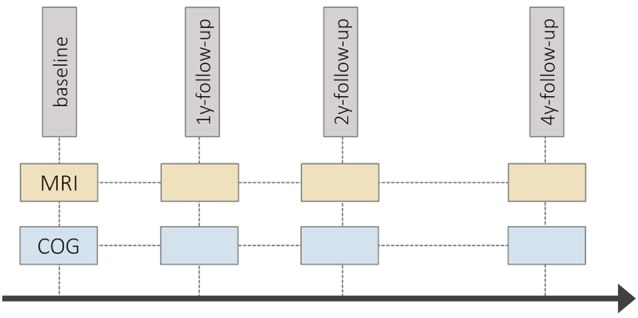
Schematic diagram of the Longitudinal Healthy Aging Brain (LHAB) study design. y, year; COG, cognitive testing session; MRI, magnetic resonance imaging.

Longitudinal cognitive and MRI data were taken from the Longitudinal Healthy Aging Brain (LHAB) database—an ongoing project conducted at the University Research Priority Program (URPP) “Dynamics of Healthy Aging” of the University of Zurich (Zöllig et al., 2011). We used data from the first four measurement occasions (baseline, 1-year follow-up, 2-year follow-up, and 4-year follow-up; see Figure 1). The baseline dataset included 232 participants (M age = 70.84; 49.14% female). At each measurement occasion, participants completed an extensive battery of neuropsychological and psychometric cognitive tests and underwent brain imaging. Brain imaging was conducted at the University Hospital of Zurich, either after cognitive testing, or on a separate day within a span of 1–2 weeks of the cognitive assessment. Inclusion criteria for study participation were age ≥64, a score of ≥26 on the Mini Mental State Examination (MMSE; Folstein et al., 1975), right-handedness, fluent German language proficiency, and no self-report of any neurological or psychiatric disease or contraindications to MRI. The study was approved by the ethical committee of the canton of Zurich. Participation was voluntary and all participants gave written informed consent in accordance with the Declaration of Helsinki.

At baseline, all 232 participants completed the processing speed tasks, and 229 also had DW-MRI data (M age = 70.72; 49.34% female). At 4-year follow-up, the dataset still comprised 74.57% of the baseline sample (*n* = 173), of which 70.69% (*n* = 164) had complete data for both processing speed and DW-MRI measures. To avoid bias due to selective exclusion of participants with incomplete data, we use the full sample (data present for processing speed and/or DW-MRI measures) in this article. Participant characteristics of the full sample at each occasion are presented in Table 1. Supplementary Table S1 provides an overview of the number of participants in the full sample, with incomplete (processing speed or DW-MRI data only), and complete data.

**Table 1:** Participant characteristics of the full sample at baseline and at each follow-up wave.

Variable	Baseline (*n* = 232)			1-year follow-up (*n* = 210)			2-year follow-up (*n* = 197)			4-year follow-up (*n* = 173)			Total selectivity
	*n*	M	SD	*n*	M	SD	*n*	M	SD	*n*	M	SD	
Baseline age (years)	232	70.84	5.08	210	70.91	5.15	197	70.66	4.81	173	70.15	4.44	−0.14
Gender (% f)	232	49.14	-	210	48.57	-	197	46.70	-	173	46.24	-	-
Education (1–3)	225	2.23	0.86	209	2.24	0.86	195	2.23	0.87	171	2.29	0.84	0.07
Mental health	212	54.79	6.26	195	54.61	6.40	184	54.55	6.25	159	54.69	5.74	−0.02
Physical health	212	50.78	7.44	195	50.90	7.44	184	51.04	6.95	159	51.43	6.44	0.09
Head motion	229	0.24	0.15	207	0.25	0.16	190	0.27	0.17	165	0.26	0.19	0.13

*Note. f, female. Education was measured on a scale from 1 to 3 (1 = high school with or without vocational education, 2 = higher education entrance qualification, business school or university of applied sciences, or 3 = university degree). Mental and physical health scores were computed based on the SF12 questionnaire, which participants filled out at home (Ware et al., 1996). Total selectivity was computed for the baseline sample as compared to the sample at 4-year follow-up (M_4-y_ȓM_base_)/SD_base_*.

To estimate whether attrition was selective, we compared the full sample at baseline with those participants still participating in the study at 4-year follow-up in these measures. For this purpose, we computed total selectivity by standardizing the difference between the mean in the baseline and the 4-year follow-up sample on the standard deviation of the baseline sample in the variable of interest (see Lindenberger et al., 2002). The size of the resulting selectivity index can be interpreted in terms of an effect size. As can be seen in Table 1, total selectivity was negligible for all measures (i.e., below the cut-off of 0.20 for a weak effect according to Cohen, 1977), suggesting that the participants remaining in the study at the 4-year follow-up did not differ from the baseline sample in terms of baseline age, education, physical and mental health, or head motion in the scanner.

### Brain Measures

#### MR Imaging

MRI measurements were conducted on a Philips Ingenia 3T scanner equipped with a commercial 32-element sensitivity encoding (SENSE) head coil array. The DW-MRI protocol employed an echo-planar (EPI) sequence [TR = 23.918 s, TE = 55 ms, FoV = 224 × 224 mm, acquisition matrix = 112 × 112, slice thickness = 2 mm, 75 contiguous slices, 2 mm^3^ isotropic voxel, flip angle = 90°, Echo Train Length (ETL) = 59, NSA = 1, SENSE factor *R* = 2.0]. One non-weighted image (*b*-value = 0 s/mm^2^) and 32 diffusion-weighted images (*b*-value of 1,000 s/mm^2^) were acquired. The diffusion-weighted directions were equally distributed in space. The same scanner and sequence was used at all measurement occasions.

#### MRI Data Preprocessing

To facilitate analysis, data were organized according to the brain imaging data structure (BIDS; Gorgolewski et al., 2016). Diffusion data was processed with a nipype pipeline (v0.14.0; Gorgolewski et al., 2011) using tools from MRtrix (3-rc2; Tournier et al., 2012), FSL (v5.0.9; Jenkinson et al., 2012), and ANTs (2.1.0; Avants et al., 2011). The analysis code is publicly available at https://github.com/fliem/extract_FA, and a BIDS-Apps-compatible (Gorgolewski et al., 2017) software container to reproduce the analysis can be found here at http://hub.docker.com/r/fliem/extract_fa/.

**Figure 2:**
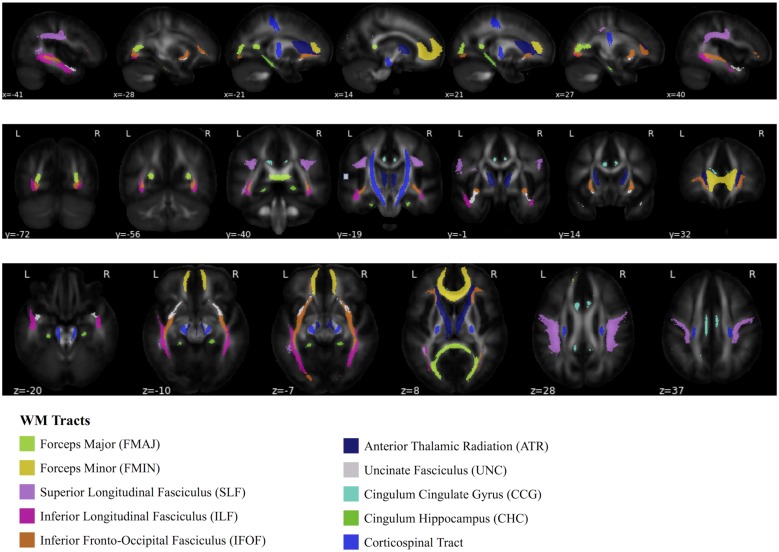
Depiction of 10 white matter (WM) tracts, based on the Johns Hopkins University WM tractography atlas (Hua et al., 2008).

The diffusion data were de-noised (Veraart et al., 2016a,b) and corrected for eddy current distortions and head motion (Andersson and Sotiropoulos, 2016; Andersson et al., 2016). Subsequently, the data were bias-corrected (Tustison et al., 2010) and a WM mask was created (Dhollander et al., 2016). Tensor maps were calculated (Veraart et al., 2013) and FA maps were derived (Basser et al., 1994; Westin et al., 1997). ANTs was used to register FA maps to the JHU-ICBM-FA template (included in FSL). Mean FA was extracted for tracts of the JHU white-matter tractography atlas (thresholded at 25% probability) for voxels with FA > 0.2 (Hua et al., 2008). The tracts considered here are: forceps major (FMAJ), forceps minor (FMIN), left and right hemispheric superior longitudinal fasciculi (SLF), inferior longitudinal fasciculi (ILF), inferior fronto-occipital fasciculi (IFOF), anterior thalamic radiations (ATR), uncinate fasciculi (UNC), cingulum cingulate gyri (CCG), cingulum hippocampus (CHC), and corticospinal tracts (CST). A graphical depiction of these tracts is shown in Figure 2. Since we did not have any specific hypothesis of lateralized aging-effects in FA, we averaged left and right hemispheric tracts, weighted by the number of voxels of the respective tract, to obtain 10 dependent variables that entered the statistical analyses.

Correlations across measurement occasions were high for all WM tracts (*r* = 0.65–0.96), indicating good reliability of FA estimates across time. Descriptive statistics, and correlations within and across measurement occasions for the raw FA values for all WM tracts can be found in the Supplementary Material (Supplementary Table S2). Longitudinal plots of intra-individual FA changes in each of the WM tracts can be found in the Supplementary Material (Supplementary Figure S1). For the statistical analyses, we multiplied FA values by 100, in order to avoid potential artifacts in bivariate model estimation due to scaling differences.

#### Head Motion Control

As a means of ensuring sufficient quality of the data, we removed FA values for 56 individual observations (i.e., 6.03% of the total N of 928 observations) at which participants showed excessive head motion. As a measure of head motion, we used the summary statistic of average RMS motion as compared to the previous slice in a volume, which was calculated during preprocessing (Andersson and Sotiropoulos, 2016). Excessive values were defined as any value more than three median absolute deviations (MADs) above the median of the sample distribution across measurement occasions (Leys et al., 2013). We used the median as a reference since it is more robust to the influence of extreme values than the mean.

### Cognitive Ability Measures

Processing speed was assessed by four psychometric paper-pencil tests described in detail below. Correlations across measurement occasions were relatively high for all tests (*r* = 0.51–0.84), indicating good reliability of these measures across time. Descriptive statistics, and correlations within and across measurement occasions for the raw processing speed values for all tasks can be found in the Supplementary Table S3. Longitudinal plots of intra-individual changes in each of the processing speed tasks can be found in the Supplementary Material (Supplementary Figure S2).

#### Identical Pictures Test

The Identical Pictures Test (IPT) is a sub-test of the Kit of Factor-Referenced Cognitive Tests (KIT; Ekstrom et al., 1976), and consists of two parts with 48 items each. For each item, participants had to distinguish which of five alternative images was identical to a target image and mark the respective one with a pen. Participants had a time limit of 90 s for each test part. Ability was measured as the number of correct responses across both test parts.

#### Digit Symbol Test

The Digit Symbol Test (DIGSY) is part of the Wechsler Intelligence Scale for Adults (Von Aster et al., 2006). The test consisted of several rows of digits ranging from 1 to 9. Participants were required to copy, from left to right, abstract symbols below the corresponding digits. A translation key on the top of the test sheet indicated which symbol was uniquely paired with which digit and was visible during the entire time of testing. Ability was measured as the number of correct responses within 2 min.

#### Trail-Making-Test A

The Trail-Making-Test A (TMTA; Reitan and Wolfson, 2004) required participants to connect numbers from 1 to 25 (in ascending order) with a pen as quickly as possible. A test instructor ensured that participants did not detach the pen from the paper during the test. If an error was made, the test instructor advised the participant to go back to the last number before the error occurred. Ability was the total time in seconds needed to perform this task (including the time used for error prompting and correction), multiplied by −1 so that higher scores equaled better performance.

#### LPS14 Test

The Leistungsprüfsystem (LPS) 14 is a sub-test of the LPS 50+, which is an adapted version of the LPS for people aged between 50 and 90 years (Sturm et al., 1993). The LPS14 sub-test consisted of two columns of letter and digit strings printed next to each other. Participants were asked to compare the two columns row-by-row and cross out the letters or digits in the row of the right column that was not identical to the letter or digit printed in the same position in the left column. Ability was measured as the number of correct responses within 2 min.

### Covariates

To control for potential confounding influences, we included age at baseline (Age_base_), level of education (on a scale from 1 to 3; 1 = high school with or without vocational education, 2 = higher education entrance qualification, business school or university of applied sciences, or 3 = university degree) and gender (0 = female, 1 = male) as covariates on the intercept and slope terms into all statistical analyses. Furthermore, for the FA models, we also included head motion in the scanner as a time-varying covariate on the manifest indicators at each measurement occasion. To facilitate model interpretation, age was centered at 70 years (median of the sample), and education at level 2. Head motion was left un-centered, since a value of zero was meaningful (i.e., reflecting no head motion).

### Statistical Analysis

All statistical analyses were run in R version 3.3.3 (R Core Team, 2019). Outlier correction in each processing speed measure was done using a cut-off of three MADs above or below the median of the sample distribution across measurement occasions, resulting in the removal of 32 individual values (*n* = 2 for DIGSY; *n* = 4 for LPS14: *n* = 4 for IPT: *n* = 22 for TMTA). We refrained from outlier control in FA measures, since FA can largely vary between individuals (e.g., Veenith et al., 2013), and no clear consensus exists on normative cut-offs. However, we excluded individual observations with excessive head motion values (see “Head Motion Control” section above).

#### Structural Equation Modeling (SEM)

To model inter-individual differences in intra-individual change in FA and processing speed, and cross-domain interactions between these variables, we estimated univariate and bivariate LCS models in the structural equation modeling (SEM) framework using the *lavaan* package version 0.5–23.1097 (Rosseel, 2012) in R. As is the standard in longitudinal SEM modeling, we treated missing values as missing at random (MAR; Little, 1995) and retained them in the model by using the full information maximum likelihood estimation (FIML; Finkbeiner, 1979; Schafer and Graham, 2002) to deal with incomplete data.

##### Univariate Models

**Figure 3:**
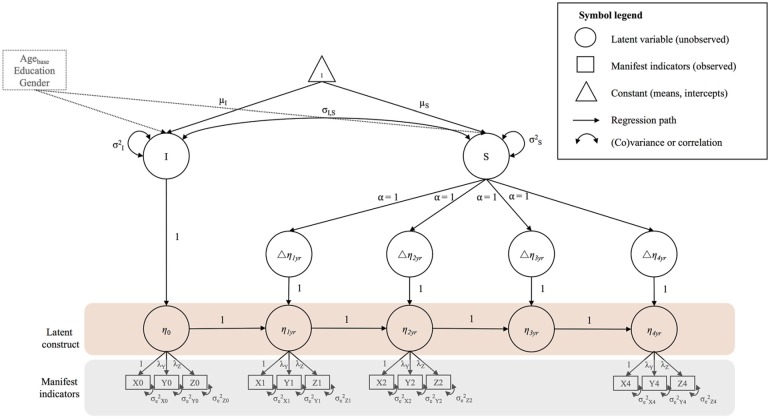
Example diagram of a univariate second-order latent change score (LCS) model. For a detailed description see “Materials and Methods” section (univariate models). All unlabeled paths are fixed to 1. Parameters with the same label are fixed to be equal. Strong measurement invariance (MI) is imposed on the model by fixing the factor loadings and intercepts of the manifest indicators to be equal over time. The manifest indicator intercepts are not shown for visual clarity. Correlated residuals of the same manifest indicator over time were estimated, but are also not shown for visual clarity. Intercept and slope variance are controlled for age at baseline (Age_base_), education and gender.

One aim of the present article was to investigate whether a global FA factor capturing the shared variance across 10 WM tracts would be a good fit to the data. Furthermore, we intended to measure processing speed on the ability level. Therefore, we used second-order LCS models (McArdle, 2009; for tutorials see Ghisletta and McArdle, 2012; Kievit et al., 2018) to estimate initial values, and 4-year changes in latent factors of FA and processing speed. Figure 3 shows a path diagram of a second-order LCS model with a latent construct (*η*_0_…*η*_4yr_; area shaded in pink) measured by three manifest, i.e., observed, indicators (X, Y, Z; area shaded in gray) at each measurement occasion. A minimum of three indicators is needed to identify a latent construct without using information from other parts of the model (Bontempo et al., 2012). Applying this model to processing speed, four indicators reflect performance in the four cognitive tasks. For FA, we extended the model in Figure 3 to include 10 indicators, reflecting FA in the 10 WM tracts. To create equal intervals between measurement occasions, we included a latent placeholder variable (*η*_3yr_) between the 2-year (*η*_2yr_) and the 4-year (*η*_4yr_) follow-up (Little, 2013). LCS were estimated between subsequent measurement occasions Δ*η*_1yr_…Δ*η*_4yr_). Consequently, a latent true score at measurement occasion t (e.g., *η*_1yr_) was perfectly explained by the latent true score at occasion t-1 (e.g., *η*_0_) and the LCS between t-1 and t (e.g., Δ*η*_1yr_). Since our study included more than two measurement occasions, we were able to estimate a latent intercept (*I*) and slope factor (*S*) on top of the LCS, to capture initial levels and overall change across time. The means of these factors reflect the average baseline value (*μ*_I_) and change (*μ*_S_) in a variable across the entire sample (i.e., fixed effects). In addition, the variances of these latent factors reflect the variability between persons (i.e., random effects) in their individual baseline values (*σ*^2^_I_) and change trajectories (*σ*^2^_S_). We fixed the loadings of the change slope (α) to a value of one, representing constant, linear change across time. The univariate LCS models estimated here can be simplified to the more commonly known *latent growth curve model*, and both models produce the same parameter estimates and fit statistics (see Grimm et al., 2016)^1^.

##### Global vs. Regional FA Changes

While a second-order LCS model estimating a latent construct with multiple indicators was acceptable for processing speed, this was not the case for FA. Therefore, we proceeded by estimating first-order LCS models for FA in each WM tract. These models were similar to the second-order model shown in Figure 3, with the difference that they were simplified to include only one manifest indicator, namely the mean of FA in the left and right homologous tract, weighted by the respective number of voxels.

##### Measurement Invariance

To assure that we measured the same construct at each measurement occasion (Meredith, 1993; Meredith and Teresi, 2006), we assumed strong measurement invariance (MI) for the latent FA and processing speed constructs. This is achieved by constraining both the loadings of the observed indicators on the latent factor (weak MI), and the indicator intercepts to equality across measurement occasions (strong MI; Widaman and Conger, 2010). By comparing these models to a model where the respective parameters vary freely (configural MI), it is then possible to test in a step-wise fashion (i.e., comparing the configural to a weak MI model, and the latter to a strong MI model), which level of MI is supported by the data (Putnick and Bornstein, 2016). Based on previous recommendations, we accepted a model with higher MI to hold over a model with lower MI if the drop in the CFI was equal to or below Δ0.01 (see Cheung and Rensvold, 2002; Chen, 2007).

##### Retest Effects

One assumption that is often made in the LCS model is that the modeled changes entirely reflect the change process of interest, i.e., aging due to the passing of time. In case of the model depicted in Figure 3, this is implemented by setting the means and residual variances of the LCS (Δη_1yr…Δη4yr_) to zero, such that change is fully captured by the constant change slope (S). However, it is well-known that participant’s performance in cognitive tests can be influenced by other factors, such as performance anxiety or unfamiliarity with the testing material (Hoffman et al., 2011). In the present study, participants were confronted with the testing situation and material for the first time at the baseline assessment, thus it is very likely that the change in processing speed between baseline and 1-year follow-up is due to both, “real change” as well as increased familiarity with the situation. To probe whether processing speed was affected by such retest effects, we additionally fit two models where either only the mean or both the mean and error variance of the first LCS were allowed to vary freely (see Barker et al., 2014).

##### Bivariate Models

**Figure 4:**
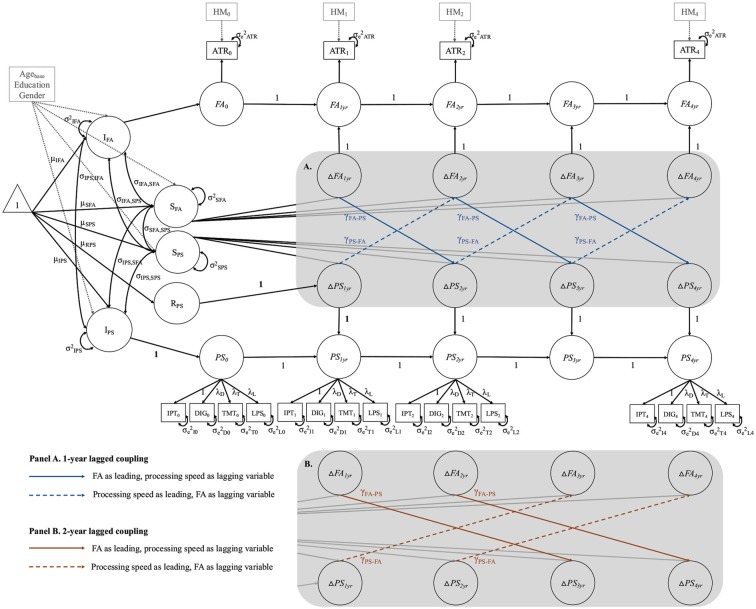
Schematic diagram of the full-coupling bivariate LCS model, combining the two best-fitting univariate LCS models for fractional anisotropy (FA) in the respective WM tract (using the anterior thalamic radiations (ATR) as example), and processing speed (PS). The gray-shaded area contains bidirectional cross-domain couplings between changes in FA and changes in PS for the 1-year lagged model series **(A)**, and for the 2-year lagged model series **(B)**. For a detailed description see “Materials and Methods” section (bivariate models). DIG, Digit Symbol task; TMT, Trail Making Test A; LPS, Leistungsprüfsystem 14; yr, year; R_PS_, retest effect between baseline and 1-year follow-up. FA is measured as first-order LCS model, and PS estimated as second-order LCS model, with a latent construct (PS_0_…PS_4yr_) at each measurement occasion, comprising four manifest indicators. Strong MI is imposed on the PS model by setting the factor loadings and intercepts of the manifest indicators equal over time. The manifest indicator intercepts are not shown for visual clarity. Correlated residuals of the same manifest indicator over time were estimated, but are also not shown for visual clarity. Squares represent observed variables, and circles represent latent variables. One-headed arrows stand for regression paths and two-headed arrows for variances and covariances. The triangle represents means and intercepts. All unlabeled paths are fixed to 1. Parameters with the same label are fixed to be equal. Intercept and slope of FA (I_FA_, S_FA_) and PS (I_PS_, S_PS_) are controlled for age at baseline (Age_base_), education and gender. Manifest FA scores were adjusted for head motion (HM_0_ …HM_4yr_) at each measurement occasion.

To estimate cross-domain relations between FA and processing speed, we combined the best fitting univariate LCS models for both domains into a *bivariate LCS model*^2^ (see Figure 4). In a first step, we estimated a *baseline model* that only included cross-domain correlations between intercepts and slopes, thus estimating static between-person associations between these two domains at baseline, and across time. As can be seen in Figure 4, this included intercept-intercept (σ_IPS, IFA_), slope-slope (σ_SFA, SPS_), and intercept-slope correlations (σ_IFA, SPS_ and σ_IPS, SFA_) among FA and processing speed. In the next step, we fit two series of models to test the two hypotheses of 1-year and 2-year lagged change-change relationships between FA and processing speed. For each series, we estimated three models that differed from the baseline model only by the inclusion of cross-lagged regression paths, so-called coupling parameters, between changes in the two domains. In Figure 4A, the cross-lagged paths for the 1-year lagged model series are shown in blue font. In Figure 4B, the cross-lagged paths for the 2-year lagged model series are shown in orange font. The first two models included a unidirectional coupling parameter, assuming either FA as leading, and processing speed as lagging variable (γ_FA-PS_), or vice versa (γ_PS-FA_). Finally, in the *full coupling model*, we included both coupling parameters, estimating bidirectional change-change associations. Hence, all three models estimated dynamic within-person couplings between changes in one domain as a leading indicator of changes in the other domain, or both, while controlling for static between-person associations between these domains. We fixed the coupling parameters to be equal over time in the respective models, thus assuming similar effects over the study period.

##### Evaluation of Model Fit

Overall model fit was evaluated by the *χ*^2^ test, specifically, by the ratio of the *χ*^2^ test statistic to the respective degrees of freedom (Jöreskog and Sörbom, 1993). Furthermore, the Comparative Fit Index (CFI; Bentler, 1990), and the root mean square error of approximation (RMSEA; Steiger and Lind, 1980) were used to evaluate goodness-of-fit. Good model fit was defined as a ratio of *χ*^2^/*df* ≤ 2, CFI > 0.97, RMSEA ≤ 0.05, and adequate fit was defined as *χ*^2^/*df* ≤ 3, CFI > 0.95, RMSEA between 0.05 and 0.08 (see Jöreskog and Sörbom, 1993; Schermelleh-Engel et al., 2003). Models were compared using the difference *χ*^2^ test (for nested models) and the sample size adjusted Bayesian Information Criterion (BIC; Raftery, 1995). The BIC is not interpretable in isolation, however, in model comparisons, smaller values indicate a closer fit of the model to the data (Kass and Raftery, 1995; Raftery, 1995). For the difference *χ*^2^ test, we lowered the significance threshold to *p* < 0.01 to reduce the likelihood of Type I error.

## Results

### Univariate Models: FA

In a first step, we evaluated whether a global FA factor capturing the shared variance across the WM tracts of interest was tenable. Therefore, we fit a univariate LCS model with a global FA factor based on 10 indicators and strong MI. This model had unacceptable fit for the data [*χ^2^*_(851)_ = 4,046.392, *χ*^2^/*df* = 4.75, RMSEA = 0.127 (0.123–0.131), CFI = 0.775, BIC = 26,064.112]. Freeing the intercepts led to a substantial increase of the CFI (ΔCFI = 0.024), suggesting that strong MI was not supported for this global FA model. Closer investigation of modification indices revealed that the intercepts of multiple indicators (i.e., multiple WM tracts) were not invariant. Even when freeing constraints on both intercepts and factor loadings (configural invariance), model fit remained unacceptable across all fit indicators [*χ*^2^_(797)_ = 3,584.301, *χ*^2^/*df* = 4.50, RMSEA = 0.123 (0.119–0.127), CFI = 0.804, BIC = 25,724.994]. We also tried to fit a reduced model that included only those WM tracts with sufficient variance in change (FMIN, IFOF, UNC, SLF, ATR; see results below). However, this model still did not yield acceptable fit [*χ*^2^_(221)_ = 1,376.497, *χ*^2^/*df* = 6.23, RMSEA = 0.150 (0.143–0.158), CFI = 0.836, BIC = 13,112.579]. Thus, we concluded that a global FA factor was not tenable in the present dataset. Hence, tract-specific models should be evaluated.

**Figure 5:**
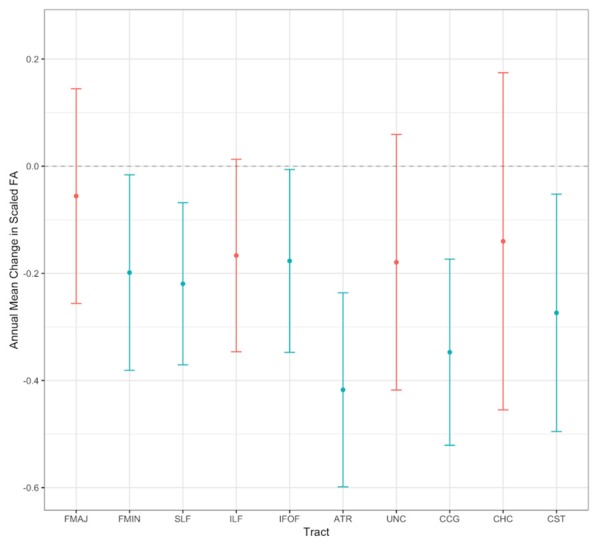
Visualization of model-predicted annual mean changes in scaled FA for all WM tracts. Error bars show 95% confidence intervals. Non-significant annual mean changes are printed in red, and significant values in blue (*p* < 0.05). See Supplementary Table S4 for the exact parameter estimates.

In a second step, we investigated how FA changes in the WM tracts of interest over time. For this purpose, we estimated separate LCS models for each WM tract. These models all showed adequate to good model fit across the majority of fit indicators (*χ*^2^_(26)_ = 34.428–77.611, *χ*^2^/*df* = 1.32–2.99, RMSEA = 0.037–0.092, CFI = 0.953–0.992), except for the CHC. The CHC and the CST initially converged with a negative slope variance, most probably due to the small variability between individuals’ change trajectories. One possible solution is to fix such negative variances to zero (for an extended discussion, see Dillon et al., 1987), as we did in the present case, which resulted in an acceptable model fit for the CST, but not for the CHC (i.e., RMSEA = 0.087, CFI = 0.912). Hence, the results for the CHC are not reliable. Scaled mean FA at baseline ranged from 35.796 in the SLF to 55.536 in the FMAJ^3^. Significant annual mean declines were observed for the FMIN, SLF, IFOF, ATR, CCG, and CST (ranging from unstandardized estimates of −0.177 to −0.417). In contrast, the FMAJ, ILF, UNC and CHC did not show significant mean changes over time. Annual mean changes in scaled FA for all tracts are also visually shown in Figure 5 (for detailed results of these models and model fit statistics see Supplementary Table S4). Baseline FA was not significantly correlated with change over time for any of the tracts, indicating that participants with higher FA values at baseline did not change differently from participants with lower FA values. Third, independent of mean change, we examined whether individuals varied substantially in their change trajectories over the study period. We found significant between-person variance in baseline FA values for all tracts, indicating that participants varied substantially in their initial FA values across all tracts. Furthermore, we found that five tracts also showed significant or marginally significant (i.e., *p* = 0.052) between-person variance in change slopes (FMIN, SLF, IFOF, ATR, UNC), indicating that participants differed substantially from each other with regard to FA changes in these tracts. We retained only the latter tracts for the subsequent bivariate analyses with processing speed since sufficient variance in change is necessary in order to analyze longitudinal bivariate associations (Raz et al., 2008; Lövdén et al., 2014).

Finally, we investigated whether covariates had an impact on baseline FA values or changes therein across 4 years. The effects of the time-invariant covariates (age, gender, education) on FA at baseline and across time are presented in Supplementary Table S5. The effects of head motion on FA at each time point are shown in Supplementary Table S6. We found that older age was associated with significantly lower baseline FA values (except for the CHC and CST), and steeper annual declines (except for the SLF, ILF, and CHC). The size of cross-sectional age-effects was larger than that of the longitudinal effects. Education had no impact on FA at baseline or changes across time in any of the WM tracts. Effects of gender on baseline FA were limited to the FMIN (−0.592, *SE* = 0.299, *p* = 0.048) and CHC (0.794, *SE* = 0.388, *p* = 0.041), and effects of gender on changes in FA were limited to the ATR (0.151, *SE* = 0.047, *p* = 0.001) and the CST (0.233, *SE* = 0.057, *p* < 0.001): male participants had on average different baseline FA (lower in the FMIN and higher in the CHC) or less FA declines in these tracts than females. Most tracts were impacted by head motion, such that more motion in the scanner was associated with significantly lower FA estimates. Please note that one unit increase in head motion amounts to almost five times the average head motion present in the sample (from 0.24 to 0.27 across measurement occasions; see Table 1), thus, the effects in Supplementary Table S6 need to be interpreted as effects of excessive motion.

### Univariate Models: Processing Speed

Comparable to the global FA factor for the WM tracts, we first investigated whether a latent processing speed variable based on the shared variance across four processing speed tasks was a good representation of the data. For this purpose, we fit a univariate LCS model with a latent processing speed factor based on four indicators and strong MI. This model had acceptable fit for all fit measures except for the CFI [*χ*^2^_(143)_ = 314.551, *χ*^2^/*df* = 2.20, RMSEA = 0.072 (0.061–0.083), CFI = 0.940, BIC = 22,709.758]. MI testing revealed a model with weak MI to be superior (ΔCFI = 0.022)^4^. After the inspection of modification indices, we identified the intercepts of the LPS14 as the source of non-invariance. Freeing these intercepts led to an improvement in model fit, such that a model with partial-strong MI did not differ substantially from a model with weak MI (ΔCFI = 0.007). The model with partial-strong MI provides very similar results for the analyses presented in this article, which is why we retain the model with full-strong MI.

In a second step, we probed whether the initial unfamiliarity with the testing situation led to retest effects between baseline and 1-year follow-up. Therefore, we fit a model where we freely estimated the mean of the first LCS. Freely estimating the mean of this but not the other change scores contains the assumption that change between baseline and 1-year follow-up is not entirely reflecting “true change” over time (as captured by the slope parameter), but is additionally influenced by other variables, such as repeated testing. This model showed adequate fit for all indicators [*χ^2^*_(142)_ = 262.879, *χ*^2^/*df* = 1.85, RMSEA = 0.061 (0.049–0.072), CFI = 0.957, BIC = 22,660.363], supporting the presence of retest effects. However, additionally freeing the variance of the first LCS resulted in an improper solution (i.e., the estimation of a negative variance of the first LCS), suggesting that participants did not seem to vary with respect to retest effects. Thus, we kept the retest variance fixed to zero. Since this model showed superior fit compared to the initial model without retest (Δ*χ*^2^ = 51.672, Δ*df* = 1, *p* < 0.001), we retained it for the bivariate analyses with WM FA.

In a third step, we examined the average trajectory of change in processing speed across 4 years as well as whether participants showed substantial inter-individual variance at baseline and around this mean trajectory. At baseline, participants had on average initial mean processing speed levels of 48.459 (*SE* = 1.453, *p* < 0.001). Besides an initial increase between baseline and 1-year follow-up captured by the retest effect (2.032, *SE* = 0.343, *p* < 0.001), processing speed declined on average over time (−0.567 per year, *SE* = 0.280, *p* = 0.043). Furthermore, between-person variance with respect to both intercepts and slopes was significant, suggesting that participants showed differences in baseline levels and changes over time. Baseline processing speed was not significantly correlated with change over time (−0.491, *SE* = 0.583, *p* = 0.399), indicating that participants with higher processing speed performance at baseline did not change differently from participants with lower performance.

Finally, we investigated whether covariates influenced baseline processing speed or processing speed changes across 4 years. Age at baseline had a significant impact on both intercept and slope, such that with every 1-year increase in age, participants had −0.645 lower processing speed at baseline (*SE* = 0.096, *p* < 0.001), and showed steeper decline (−0.049 per year, *SE* = 0.017, *p* = 0.004) over time. Participants with higher education levels showed higher levels of processing speed performance at baseline (1.246, *SE* = 0.554, *p* = 0.025), but education had no effect on changes in processing speed over time (0.069, *SE* = 0.089, *p* = 0.436). No effects of gender on either intercept or slope were found. The detailed results of this model, including the effects of the time-invariant covariates on processing speed at baseline and across time are presented in Supplementary Table S7.

### Bivariate Models: FA and Processing Speed

After detecting that five WM tracts (FMIN, SLF, IFOF, ATR, UNC) show between-person variance in change slopes, we restrict bivariate models for the investigation of the relationship between change in FA and processing speed to these tracts. Specifically, we were interested in investigating lagged relationships between preceding changes in FA and subsequent changes in processing speed: (a) 1 year; or (b) 2 years later. As an alternative hypothesis, we also investigated the other directionality (changes in processing speed as a leading indicator of subsequent changes in FA) as well as bidirectional relationships between those domains. To do so, we estimated a baseline model and compared it to two series of models that estimated either unidirectional or bidirectional cross-domain couplings. The first model series was fit with 1-year time-lags, and the second series with 2-year time-lags between changes in FA and changes in processing speed. Hence, for each combination of an individual WM tract with processing speed, we estimated overall 7 × 5 = 35 bivariate LCS models.

We performed model comparisons between the baseline and each of the lagged coupled change models using *χ*^2^ difference tests since the latter models were nested within the former. Model fits for the baseline model and the coupled change models are shown in Table 2 for the 1-year time-lag, and in Table 3 for the 2-year time-lag model series. The selected model for each WM tract is shaded in gray. Please note that the baseline model was only estimated once, but is presented in both Tables. None of the 1-year lagged coupling models fit significantly better than the baseline model. However, the 2-year lagged coupling models were a better fit for the SLF and the ATR. For the SLF, both unidirectional models, and the full coupling model fit significantly better than the baseline model. Since the full coupling model also had the lowest BIC, we chose it as the best fitting model for the SLF. For the ATR, the unidirectional coupling model with FA as leading and processing speed as lagging indicator, and the full coupling model fit significantly better than the baseline model. Although the unidirectional coupling model had a slightly lower BIC, we decided to report the full coupling model here to allow better comparability with the results for the SLF. For the FMIN, IFOF and UNC, however, none of the lagged change models showed superior fit to the baseline model, which is why we retained this more parsimonious model for these WM tracts. Table 4 shows the results of the best-fitting models for these five tracts. As can be seen from this Table, none of the bivariate correlations between FA and processing speed (intercept-intercept, intercept-slope, slope-slope) reached significance in the baseline models of FMIN, IFOF, and UNC, indicating an absence of static cross-domain associations in these tracts. With respect to the 2-year lagged change models for the SLF and ATR, significant positive cross-sectional correlations were found between FA values and processing speed at baseline, indicating that participants with higher FA values showed better processing speed. These effects were small to typical in effect size (fully standardized estimate = 0.151 for SLF, and 0.198 for ATR) as compared to the standards in inter-individual difference research (Gignac and Szodorai, 2016). Of specific interest were the results for the 2-year lagged dynamic coupling effects from FA to processing speed. This effect was significant and positive for both the SLF (2.570, *SE* = 1.119, *p* = 0.022) and the ATR (3.030, SE = 1.265, *p* = 0.017), indicating that less intra-individual declines in FA were related to fewer declines in processing speed 2 years later. Moreover, for the SLF, we also found a positive effect of preceding changes in processing speed on subsequent changes in FA (0.607, *SE* = 0.229, *p* = 0.008). This effect was smaller than the coupling effect from FA to processing speed and reached significance due to the smaller standard error. No effect of changes in processing speed on subsequent changes in FA were found for the ATR. Since previous MI testing revealed that strong MI is not fully supported for the processing speed part of the model, we re-calculated the results for the best-fitting bivariate models, but now assuming only partial-strong MI (i.e., by freeing the intercepts of the LPS14 indicators). All results remained largely similar, with one exception: While the 2-year lagged dynamic coupling effect from FA to processing speed remained significant and positive for both the SLF (2.854, *SE* = 1.387, *p* = 0.040) and the ATR (3.251, *SE* = 1.292, *p* = 0.012), the reverse coupling effect from processing speed to FA for the SLF did no longer reach significance (0.420, *SE* = 0.248, *p* = 0.091), and should, therefore, be interpreted with caution.

**Table 2:** Model fit indices from bivariate LCS models of FA in five WM tracts with processing speed (1-year coupling).

		Model fit					Model comparison
Tract		*χ*^2^ (*df*)	*χ*^2^/*df*	RMSEA (95% *CI*)	CFI	BIC	Δχ^2^ (Δ*df*)
FMIN	Baseline model	427.070 (292)	1.46	0.045 (0.035–0.054)	0.966	24,189.362	-
	FA → PS	427.029 (291)	1.47	0.045 (0.035–0.054)	0.966	24,191.598	0.041 (1)
	PS → FA	425.066 (291)	1.46	0.045 (0.035–0.053)	0.966	24,189.635	2.004 (1)
	Full coupling	424.975 (290)	1.47	0.045 (0.035–0.054)	0.966	24,191.821	2.095 (2)
IFOF	Baseline model	427.278 (292)	1.46	0.045 (0.035–0.054)	0.965	24,214.512	-
	FA → PS	427.239 (291)	1.47	0.045 (0.035–0.054)	0.965	24,216.752	0.039 (1)
	PS → FA	425.303 (291)	1.46	0.045 (0.035–0.054)	0.965	24,214.815	1.975 (1)
	Full coupling	425.090 (290)	1.47	0.045 (0.035–0.054)	0.965	24,216.880	2.188 (2)
UNC	Baseline model	457.964 (292)	1.57	0.049 (0.041–0.058)	0.954	24,481.152	-
	FA → PS	457.961 (291)	1.57	0.050 (0.041–0.058)	0.954	24,483.426	0.003 (1)
	PS → FA	457.907 (291)	1.57	0.050 (0.041–0.058)	0.954	24,483.372	0.057 (1)
	Full coupling	457.750 (290)	1.58	0.050 (0.041–0.058)	0.953	24,485.493	0.214 (2)
SLF	Baseline model	471.856 (292)	1.62	0.052 (0.043–0.060)	0.954	23,951.350	-
	FA → PS	471.601 (291)	1.62	0.052 (0.043–0.060)	0.954	23,953.371	0.255 (1)
	PS → FA	470.545 (291)	1.62	0.052 (0.043–0.060)	0.954	23,952.315	1.311 (1)
	Full coupling	470.197 (290)	1.62	0.052 (0.043–0.060)	0.954	23,954.245	1.659 (2)
ATR	Baseline model	443.327 (292)	1.52	0.047 (0.038–0.056)	0.961	24,224.647	-
	FA → PS	443.070 (291)	1.52	0.047 (0.038–0.056)	0.961	24,226.667	0.257 (1)
	PS → FA	442.035 (291)	1.52	0.047 (0.038–0.056)	0.961	24,225.632	1.292 (1)
	Full coupling	441.877 (290)	1.52	0.048 (0.038–0.056)	0.961	24,227.751	1.450 (2)

*Note. PS = processing speed. Models were compared to the baseline model. The gray shading indicates the selected model for the respective WM tract. None of the lagged change models fit significantly better than the baseline model (p < 0.01)*.

**Table 3:** Model fit indices from bivariate LCS models of FA in five WM tracts with processing speed (2 year-coupling).

		Model fit					Model comparison
Tract		*χ*^2^(*df*)	*χ*^2^/*df*	RMSEA (95% *CI*)	CFI	BIC	Δχ^2^ (Δ*df*)
FMIN	Baseline model	427.070 (292)	1.46	0.045 (0.035–0.054)	0.966	24,189.362	-
	FA → PS	424.281 (291)	1.46	0.044 (0.035–0.053)	0.966	24,188.850	2.789 (1)
	PS → FA	426.716 (291)	1.47	0.045 (0.035–0.054)	0.966	24,191.285	0.354 (1)
	Full coupling	423.427 (290)	1.46	0.045 (0.035–0.053)	0.966	24,190.273	3.643 (2)
IFOF	Baseline model	427.278 (292)	1.46	0.045 (0.035–0.054)	0.965	24,214.512	-
	FA → PS	423.071 (291)	1.45	0.044 (0.035–0.053)	0.966	24,212.583	4.207 (1)
	PS → FA	427.095 (291)	1.47	0.045 (0.035–0.054)	0.965	24,216.607	0.183 (1)
	Full coupling	422.943 (290)	1.46	0.044 (0.035–0.053)	0.966	24,214.732	4.335 (2)
UNC	Baseline model	457.964 (292)	1.57	0.049 (0.041–0.058)	0.954	24,481.152	-
	FA → PS	457.521 (291)	1.57	0.050 (0.041–0.058)	0.954	24,482.986	0.443 (1)
	PS → FA	456.255 (291)	1.57	0.049 (0.041–0.058)	0.954	24,481.719	1.709 (1)
	Full coupling	451.280 (290)	1.56	0.049 (0.040–0.058)	0.955	24,479.022	6.684 (2)
SLF	Baseline model	471.856 (292)	1.62	0.052 (0.043–0.060)	0.954	23,951.350	-
	FA → PS	464.919 (291)	1.60	0.051 (0.042–0.059)	0.956	23,946.690	**6.937 (1)**
	PS → FA	456.875 (291)	1.57	0.050 (0.041–0.058)	0.958	23,938.645	**14.981 (1)**
	Full coupling	447.505 (290)	1.54	0.048 (0.039–0.057)	0.960	23,931.552	**24.351 (2)**
ATR	Baseline model	443.327 (292)	1.52	0.047 (0.038–0.056)	0.961	24,224.647	-
	FA → PS	434.948 (291)	1.49	0.046 (0.037–0.055)	0.963	24,218.545	**8.379 (1)**
	PS → FA	443.128 (291)	1.52	0.047 (0.038–0.056)	0.961	24,226.725	0.199 (1)
	Full coupling	433.978 (290)	1.50	0.046 (0.037–0.055)	0.963	24,219.853	**9.349 (2)**

*Note. PS = processing speed. Models were compared to the baseline model. Values printed in bold were significant at least at *p* < 0.01. The gray shading indicates the selected model for the respective WM tract*.

**Table 4:** Results for best-fitting bivariate LCS models FA in five WM tracts with processing speed.

	Baseline model			Lagged full coupling model (2 years)	
Parameter estimates/WM tract	FMIN	IFOF	UNC	SLF	ATR
**Bivariate correlations**					
Intercept FA, PS	1.451 (0.972)	1.303 (0.897)	0.865 (1.002)	1.754* (0.736)	2.482** (0.854)
Intercept FA, Slope PS	0.206 (0.155)	0.137 (0.157)	0.235 (0.162)	0.170 (0.145)	0.025 (0.188)
Intercept PS, Slope FA	0.022 (0.144)	0.086 (0.136)	0.239 (0.209)	−0.024 (0.173)	0.131 (0.164)
Slope FA, Slope PS	−0.009 (0.022)	−0.024 (0.022)	−0.006 (0.028)	−0.037 (0.038)	−0.090 (0.052)
**Longitudinal coupling**					
FA → PS	-	-	-	2.570* (1.119)	3.030* (1.265)
PS → FA	-	-	-	0.607** (0.229)	0.109 (0.139)

Note. PS = processing speed. Parameter estimates are unstandardized, and adjusted for effects of age at baseline, education, and gender (on intercept and slope of both FA and PS). Manifest FA scores were additionally adjusted for head motion at each measurement occasion. Values printed in bold were significant at *p < 0.05, **p < 0.01.

## Discussion

The focus of the present study was to examine the dynamic interplay of intra-individual changes in FA of 10 major WM tracts with changes in processing speed. Specifically, we examined whether changes in FA preceded changes in processing speed by 1 or 2 years, and vice versa. In addition, we intended to characterize the nature of age-related FA changes across the investigated WM tracts, i.e., whether changes are regionally distinct or globally shared. We used LCS models to estimate inter-individual differences in intra-individual changes in FA and processing speed in a large sample of healthy older adults measured repeatedly across 4 years. We found that a model assuming a single latent FA variable at each measurement occasion resulted in a bad fit to the data. We, therefore, conclude that FA changes are not fully shared across brain-wide WM tracts, but differ depending on the WM tract under study. Investigating these regional WM tracts separately, we found significant mean declines in FA for five WM tracts (FMIN, SLF, ATR, CCG, CST). The other tracts did not show any significant mean changes over time, suggesting average stability across 4 years. Importantly, three of these tracts (FMIN, SLF, ATR) and also two of the tracts that displayed mean stability (IFOF, UNC) revealed significant between-person variability in the individual longitudinal trajectories, suggesting that in these tracts, individuals diverged substantially from the mean trend. Associating FA changes in this subset of WM tracts with changes in processing speed, we found evidence supporting an association of within-person changes in the SLF and ATR with lagged changes in processing speed 2 years, but not 1 year, later. For the SLF, we also found evidence for the reciprocal relationship of changes in processing speed as a leading indicator of changes in FA 2 years later. However, this effect was much smaller in size, and not stable when partial MI was assumed (see “Limitations and Future Directions” section for a discussion). In addition, both tracts showed positive associations between FA and processing speed at baseline, indicating that individuals with higher FA values in these tracts show higher processing speed performance. In contrast, the FMIN, IFOF, and UNC—for which we also observed significant inter-individual slope variance, did not show any bivariate associations with processing speed.

### Lagged Coupled Changes

The fact that we found support only for more distant, 2-year time lags between preceding changes in FA and subsequent changes in processing speed aligns well with the formulated theoretical models that link brain and cognitive aging. For example, the theory of cognitive reserve proposes that individuals differ in the amount of cognitive capacity, e.g., in the use of cognitive strategies or general mental flexibility, that enables them to deal with accumulating brain damage, and ultimately delay negative impacts on cognitive ability (Stern, 2002, 2009). In a similar vein, the revised Scaffolding Theory of Aging and Cognition (STAC-r) proposes that compensatory scaffolding mechanisms, e.g., *via* the recruitment of secondary functional brain networks, can buffer the immediate impacts of age-related declines in brain structure and function on cognitive abilities (Reuter-Lorenz and Park, 2014). Taken together, both theories predict that healthy aging individuals should have sufficient compensatory resources to level off brain atrophy for a certain period of time, and thus maintain cognitive ability in the short term. Consequently, if anything, only weak relations between simultaneous changes in brain connectivity and processing speed can be expected. In line with this proposition, the few existing longitudinal studies did not find a relation between changes in a measure of global (Charlton et al., 2010; Ritchie et al., 2015) or regional WM microstructure (genu and splenium of corpus callosum: Gorbach et al., 2017; fornix crus: Song et al., 2018) and simultaneous changes in processing speed. One exception is the study by Lövdén et al. (2014), who reported a significant correlation of WM microstructure changes in the corticospinal tract with changes in latent processing speed in very old adults (81–103 years) across 2 years. This association was very specific and remained after controlling for changes in the other WM tracts (FMAJ, FMIN, CCG, SLF), and in global WM or higher-order cognitive abilities (i.e., episodic memory, letter and category fluency). The authors concluded that the tasks they used to measure processing speed (i.e., digit cancellation, pattern comparison) might have required a strong motor component. At first glance, the motor component of the used tasks in the present study does not seem to differ much from the study by Lövdén et al. (2014). However, given that we only included tracts with sufficient variance in change in the bivariate models, we did not assess the relation between changes in CST and processing speed. Considering the advanced age of the participants in this study, it is also very likely that their compensatory resources were already diminished, resulting in a more immediate impact of WM degradation on processing speed.

In the present study, the finding of lagged coupled change relations between FA and processing speed was limited to specific WM tracts (ATR and SLF). And moreover, these tracts were also the only ones showing significant baseline-associations with processing speed.

First, the SLF is a long-range tract that can be anatomically separated into several distinct parts (Kamali et al., 2014). In its entirety, the SLF is connecting intra-hemispheric frontal with temporal, parietal and occipital regions (Catani et al., 2002). In empirical studies, the SLF has been related to language production (Dick and Tremblay, 2012), and visuospatial processing (Shinoura et al., 2009; Vestergaard et al., 2011). Moreover, Kerchner et al. (2012) found that FA in the SLF was associated with processing speed, independent of the age of the participants (age range 55–87 years). To measure processing speed, the authors used a composite of speeded performance in multiple visuospatial choice-reaction time tasks. In the present study, we measured processing speed with four paper-pencil tasks that all involved some aspect of visuospatial processing. For example, the TMTA required participants to connect numbers in an ascending order, and thus needed visuospatial attention to locate the respective next number in the order on the sheet of paper. It is thus possible that the SLF is specifically relevant in supporting the visuospatial processing aspects of speeded performance. Second, the ATR is an intra-hemispheric tract projecting from the anterior and dorsomedial nuclei of the thalamus to the frontal cortex (Coenen et al., 2012). The ATR has been linked to attentional control (Ge et al., 2013), and higher-order cognitive ability (Rossi et al., 2017). Specifically, cross-sectional studies with healthy older adults have associated inter-individual differences in WM microstructure of the ATR to speeded performance in part B of the TMT (MacPherson et al., 2017), and a processing speed composite of performance in the IPT, and a letter- and digit comparison task (similar to the LPS14 used here; Borghesani et al., 2013).

In addition, most comparable to the present study, Rabin et al. (2019) investigated associations between global and tract-specific WM microstructure at baseline with subsequent changes in processing speed, measured by the DIGSY and TMTA, across up to 7 years in healthy older adults (aged 63–90 years). The authors found that lower global FA was significantly related to subsequent declines in processing speed. However, tract-specific associations between FA and processing speed did not reach significance or were at least substantially diminished when controlling for a modified measure of global FA (excluding the respective tract of interest), suggesting an association of global WM microstructure beyond tract-specific relationships. The authors repeated these analyses across several other measures of WM microstructure (e.g., mean diffusivity) and found the same pattern of results, with one exception: the ATR showed an independent association with changes in processing speed, such that lower WM microstructure in this tract was associated with steeper declines in processing speed. Altogether, the present finding of lagged change associations being limited to the ATR and the SLF might reflect changes in visuospatial or attentional functioning underlying the specific tasks we used to measure processing speed. Despite that—based on previous research—the two tracts can be convincingly related to the tasks used in the present study we would refrain from over-interpreting the observed regional specificity. It could also be that the 2-year lag is only just at the lower limit to reveal lagged coupled associations between brain and behavioral changes. Accordingly, one could assume that, for the other tracts, the associations would appear more consistently with longer time lags. In addition, and consistent with this idea, the LHAB sample is very well educated compared to the Swiss population: While 15.1% of the adults in Switzerland between 65 and 74 years have a university degree, this was the case for 51.1% of the participants in the LHAB sample (Bundesamt für Statistik, 2018). Previous empirical studies have associated higher levels of education with increased cognitive reserve, and thus longer maintenance of cognitive ability (Foubert-Samier et al., 2012; Mungas et al., 2018). Partially supporting this notion, higher levels of education in the present study were associated with higher processing speed performance.

### Global vs. Regional Changes of WM Microstructure

A second aim of the present study was to investigate whether age-related FA changes are global, i.e., shared among brain-wide tracts or local, i.e., show distinct changes between regional tracts. A second-order LCS model estimating a latent FA construct at each measurement occasion, which was based on the shared variance among 10 WM tracts, did not show a satisfactory fit. Even the estimation of a reduced model that included only those WM tracts showing sufficient variance in change did not prove to be a good representation of the data. Therefore, we concluded that a global FA factor is not tenable in the present sample. The advantage of estimating latent constructs based on multiple indicators is that the respective latent variable can be evaluated for MI over time. This means that it is possible to test, whether the construct of interest is comparable, i.e., is measuring the same, across different measurement occasions. At least strong MI is needed to allow for the meaningful interpretation of changes in mean structures. In the present example, the latent FA construct, besides not fitting the data well, did not show strong MI. This means that the intercepts of the individual WM tracts changed differently than the overall construct. To our best knowledge, only three longitudinal studies have directly investigated the factor structure of WM microstructure, and evaluated MI over time (Lövdén et al., 2014; Ritchie et al., 2015; Bender et al., 2016). These studies all included two repeated assessments of WM microstructural microstructure and processing speed and covered a time span of 2–3 years. Comparable to the present study, Lövdén et al. (2014) reported an unacceptable fit of a global FA factor estimated based on five WM tracts, and testing MI further revealed that strong MI did not hold. In contrast, Bender et al. (2016) and Ritchie et al. (2015) found a global factor across multiple tracts to be acceptable. However, while Bender et al. (2016) assumed strong invariance, the authors did not report testing that assumption. Furthermore, Ritchie et al. (2015) found—similar to the present study—that strong invariance was not tenable for their global FA factor. Since this lack of invariance did not influence the main results of their analyses, however, the authors decided to retain a global FA factor. Overall, these findings tentatively suggest that age-related changes in WM microstructure unfold differently across the specific WM tracts of the brain—even though slopes are highly correlated between the tracts. To derive a final conclusion, however, is difficult due to substantial between-study differences regarding the age range of the participants, the WM tracts investigated, and the method used to extract the FA values (or other indices of WM microstructure).

### Strengths

The present study is the first to include longitudinal measurements of both WM microstructure and cognitive ability in a large sample of healthy older adults across more than two occasions. Strictly speaking, more than two occasions are needed to estimate even linear change, since a straight line always can be fit neatly through two estimates. Importantly, more than two occasions are necessary to estimate lagged relationships between developmental changes in two variables, and at least four repeated measurements—as included here—are required to estimate nonlinear trajectories of change (see King et al., 2018). Although not the primary focus of the present investigation, we also tested whether a nonlinear model of change would better represent changes in processing speed and FA (both for the global factor, and the individual tracts). Specifically, we explored, whether an extension to the linear LCS models, where previous levels were related to subsequent changes within a domain would result in improved model fit. Such an autoregressive parameter allows to model decelerating and accelerating change dynamics, in addition to the overall latent change slope. However, this extension did not show a good fit for either the processing speed or the FA models, suggesting that 4-year changes in both domains were better approximated with a linear trajectory.

Generally, the use of LCS models to estimate inter-individual differences in intra-individual change is a major strength of the present study. LCS models have the advantage that they can be flexibly adapted to test complex dynamic relations between within-person changes in two or more developmental processes. Specifically, by estimating LCS between subsequent measurement occasions, it becomes possible to estimate lagged coupled changes. To the best of our knowledge, only one study has estimated lagged couplings between changes in brain structure and changes in cognitive ability, using bivariate LCS models: in a sample of older adults (*N* = 123, age range: 60–90 years), Grimm et al. (2012) found that larger decreases in lateral ventricle volume were related to larger declines in short-term memory 1 year later.

Another advantage of LCS models, or rather of the SEM framework in general, is that it allows the estimation of latent constructs based on multiple observed indicators. In the present study, we have used this approach to estimate processing speed as a latent variable, and thus reduce the influence of task-specific measurement error. Similarly, we were also able to directly test, whether a global FA factor could capture changes in FA across multiple tracts.

Another strength of the present study is the inclusion of retest effects into the statistical model of processing speed. One of the downsides of the longitudinal design is that repeated testing can lead to confounding of actual age-related changes with retest-related gains (Salthouse et al., 2004). Specifically, if not controlled for, retest effects can lead to a positive bias of correlated change estimates between two or more variables (Ferrer et al., 2005). Here, having a longitudinal design with more than two time points again is paramount, otherwise, retest effects cannot be distinguished from effects of “real” change.

Finally, although it is well-known that head motion in the scanner can substantially bias the estimation of FA and other metrics derived from DW-MRI (Yendiki et al., 2014; Baum et al., 2018), few studies actually take the effects of head motion into consideration. In the present study, we applied a strict control for head motion, by removing observations where participants showed excessive motion in the scanner, and by entering head motion as a covariate in the statistical analyses of individual FA tracts. We found that head motion had significant effects on FA across most of the WM tracts investigated here, such that more motion was associated with lower FA values.

### Limitations and Future Directions

Even though the present dataset was of a relatively large sample size compared to other longitudinal studies that combined neuroimaging with cognitive assessments, future investigations of complex multivariate theoretical models of change, as tested here, warrant much larger sample sizes. Since longitudinal studies are time and resource-intensive, a fruitful solution to increase sample size is to pool data across multiple sites. An advantage of data pooling is that it allows researchers to test the replicability of their results in a different sample, and thus gain more insights into the generalizability of their findings (Jockwitz et al., 2019). While the present study is one of the first to include four repeated measurements of both neuroimaging and cognitive data, studies with more follow-ups and different time intervals are needed to explore the temporal dynamics of age-related changes in the brain, and their impact on subsequent changes in cognitive ability.

Furthermore, the age range in the present sample was relatively heterogeneous (64–86 years), and skewed to include more young-old than old-old participants. While a heterogeneous age range is generally informative, it can bias the estimate of change with age-related differences, especially if the metric for change is time in study. We limited confounding effects of this imbalance by controlling all of our statistical models for the participants’ age at baseline. An alternative solution would be to use age directly as the time metric. In the present study, this was not possible due to few participants in the older age categories.

While we carefully ensured to measure processing speed on a latent, ability level, and tested for longitudinal MI of this latent construct, we could only establish partial-strong MI. Our main results remained similar for the most part when we assumed strong MI in processing speed and compared it to a model with partial-strong MI. One exception was the finding of 2-year lagged couplings from preceding changes in processing speed to subsequent changes in FA of the SLF, which was no longer significant when assuming partial MI, and therefore probably unreliable. Unfortunately, it is still largely unknown what degree of violation of strong MI can be accepted in order to be able to interpret changes in mean structures (Putnick and Bornstein, 2016). Future simulation studies are urgently needed to address this question.

Given the exploratory nature of our analyses, we performed a large number of model comparisons. Therefore, we used a strict significance threshold for these comparisons (i.e., *p* < 0.01), and additionally relied on the BIC to select the best-fitting models. For the selected final models, however, we did not apply any statistical control for multiple comparisons. Little consensus exists on how best to control for multiple comparisons in complex multivariate SEM models, and hence, most researchers do not apply any Type I error control (Smith and Cribbie, 2013). One suggestion that has been made is to control for the total number of hypothesis tests performed within a model (Cribbie, 2007), however, it is still up to the researcher to decide whether this number is determined based on the structural model or also based on the measurement model. In any case, the main results presented here warrant future replication and should be interpreted with caution at the present moment.

Finally, although DW-MRI is a highly popular and sophisticated method in aging neuroscience research, the derived estimates of WM microstructure are relatively unspecific with regard to their neurobiological basis (Jones et al., 2013). Many factors of the complex WM architecture, including those unrelated to WM health (e.g., crossing or kissing fibers, water concentration) can influence tensor-derived estimates (Beaulieu, 2002; Jeurissen et al., 2013; Jones et al., 2013). Given the exploratory nature of the present study, we decided to limit our analyses to FA as an index of WM microstructure, since it is comparatively well-researched and provides a global estimate of the change in the WM fiber organization.

## Conclusion

We conclude that regional changes in WM microstructure precede changes in processing speed. Specifically, our results support theoretical predictions of more distant 2-year, but not 1-year, time-lagged change associations of WM microstructure and processing speed in some of the WM tracts under study. Furthermore, our results did not support the proposition of completely shared WM microstructure changes across the brain. Future investigations are needed to further explore the temporal dynamics between WM microstructure and processing speed.

## Data Availability Statement

The data for this manuscript are not publicly available. Since data collection was started in 2011, when public data sharing and open science were not yet widely discussed, the used consent does not allow for the public sharing of the data. We are currently working on a solution for this matter. At the moment data can only be accessed *via* collaborations within the URPP Dynamics of Healthy Aging group.

## Ethics Statement

The study was approved by the ethical committee of the canton of Zurich. Participation was voluntary and all participants gave written informed consent in accordance with the declaration of Helsinki.

## Author Contributions

SM, FL, CR, MM, and LJ contributed to the design, set-up, maintenance and support of the Longitudinal Healthy Aging Brain (LHAB) database. JO performed the statistical analysis and wrote the first draft of the manuscript. All authors contributed to manuscript revision, read and approved the submitted version.

## Conflict of Interest

The authors declare that the research was conducted in the absence of any commercial or financial relationships that could be construed as a potential conflict of interest.

## Footnotes

^1^We also tested whether adding a autoregressive parameter β between previous levels and subsequent changes of a variable would lead to improved model fit. This parameter introduces a dynamic aspect to the model, either decelerating (negative β) or accelerating (positive β) the overall change estimated by the overall slope factor, allowing for the estimation of nonlinear trajectories over time. However, this model (also known as the dual change score model; McArdle, 2001; McArdle and Hamagami, 2001, 2003; McArdle et al., 2000) was not a good fit for neither FA, nor processing speed, as determined by difference *χ*^2^ tests, problems with model convergence, or inflated parameter estimates. Therefore, we decided to proceed with the more parsimonious linear models presented here.

^2^This model is comparable to the more commonly known bivariate dual change score model (BDCSM; McArdle, 2001; McArdle et al., 2004; McArdle and Hamagami, 2001), but with the autoregressive coupling parameters set to zero, since they were not a good fit to either the FA or processing speed univariate models (see footnote 1).

^3^Divided by 100, these unstandardized estimates can be directly interpreted like raw FA values on a scale from 0 to 1. Thus, baseline FA ranged between 0.358 and 0.555.

^4^Weak MI was supported, however, as compared to a model with configural MI (ΔCFI < 0.001).
