# Supplementary material for: Lagged Coupled Changes Between White Matter Microstructure and Processing Speed in Healthy Aging: A Longitudinal Investigation
Source: Front Aging Neurosci. 2019 Nov 21;11:298. doi: 10.3389/fnagi.2019.00298 (PMC6881240; doi:10.3389/fnagi.2019.00298)
Supplement: Supplementary file 1 [file Image_1.pdf]

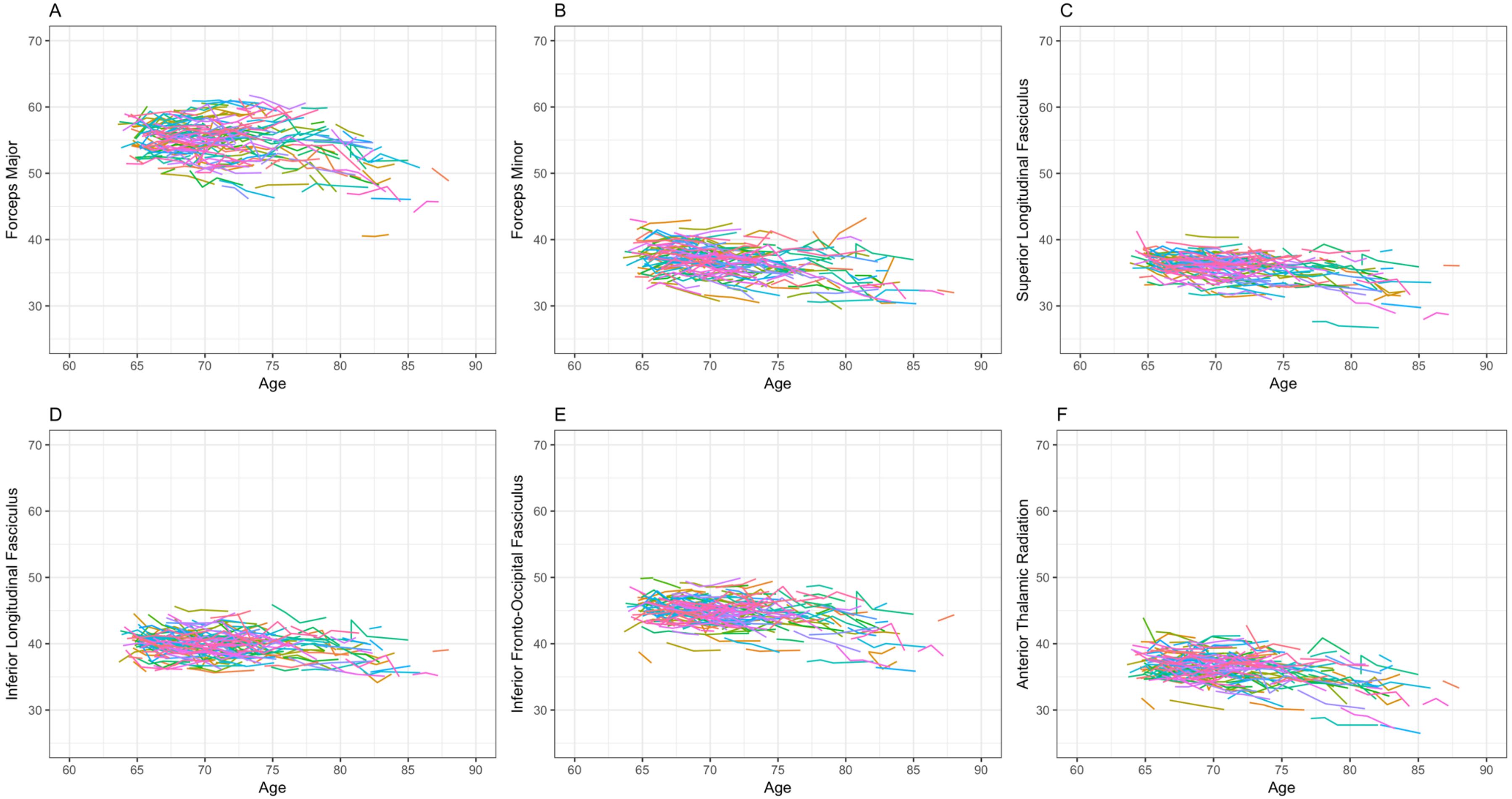

Figure S1 (part one). Spaghetti plots of 4-year changes in FA of the (A) Forceps Major (B) Forceps Minor, (C) Superior Longitudinal Fasciculus, (D) Inferior Longitudinal Fasciculus, (E) Inferior Fronto-Occipital Fasciculus, (F) Anterior Thalamic Radiation. Values for all tracts are raw FA scores, multiplied by 100.

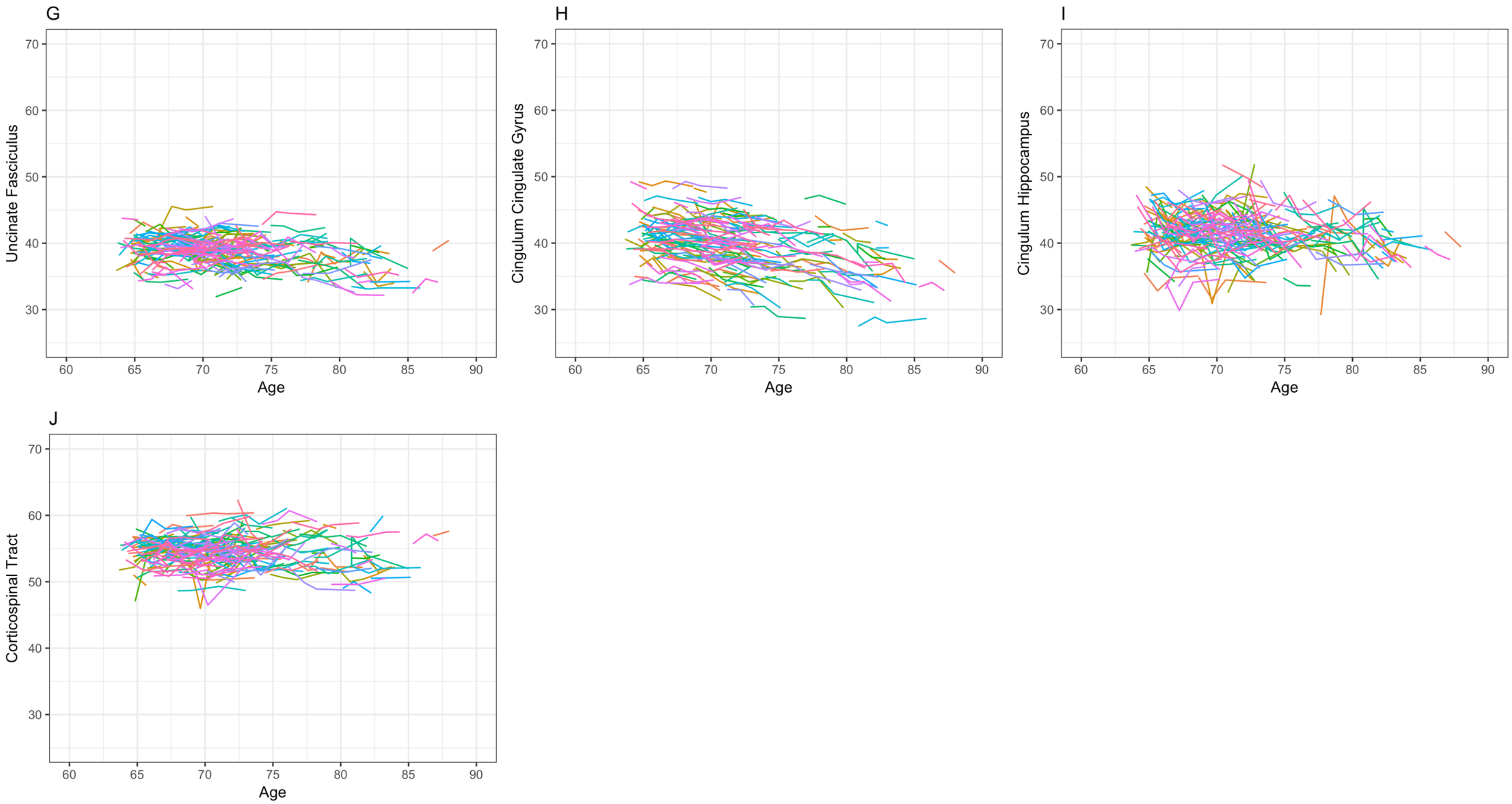

Figure S1 (part two). Spaghetti plots of 4-year changes in FA of the (G) Uncinate Fasciculus, (H) Cingulum Cingulate Gyrus, (I) Cingulum Hippocampus and (J) Corticospinal Tract. Values for all tracts are raw FA scores, multiplied by 100.
