# Supplementary material for: Lagged Coupled Changes Between White Matter Microstructure and Processing Speed in Healthy Aging: A Longitudinal Investigation
Source: Front Aging Neurosci. 2019 Nov 21;11:298. doi: 10.3389/fnagi.2019.00298 (PMC6881240; doi:10.3389/fnagi.2019.00298)
Supplement: Supplementary file 2 [file Image_2.pdf]

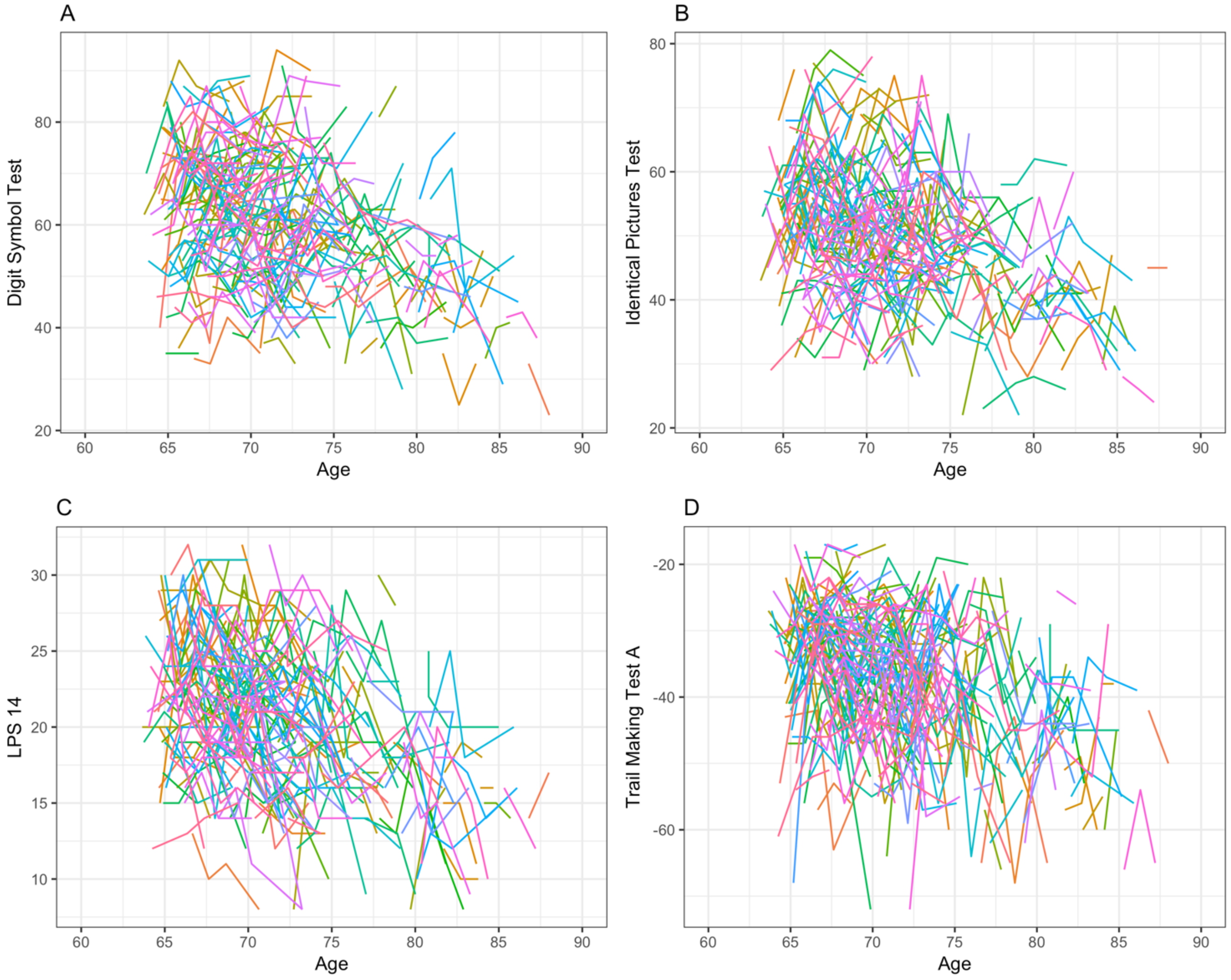

Figure S2. Spaghetti plots of 4-year processing speed changes in the (A) Digit Symbol Test (B) Identical Pictures Test, (C) Leistugsprüfsystem (LPS) 14 and (D) Trail Making Test Part A for each individual. Values for all tasks are raw scores, coded such that higher values represent better performance.
