# Supplementary material for: Lagged Coupled Changes Between White Matter Microstructure and Processing Speed in Healthy Aging: A Longitudinal Investigation
Source: Front Aging Neurosci. 2019 Nov 21;11:298. doi: 10.3389/fnagi.2019.00298 (PMC6881240; doi:10.3389/fnagi.2019.00298)
Supplement: Supplementary file 3 [file Table_1.pdf]

Table S1

Number of participants of the full sample, with data for PS or DW-MRI only, and with complete data

Variable	Baseline	1-y follow-up	2-y follow-up	4-y follow-up
	n	*n*	*n*	*n*
Full sample	232	210	197	173
Incomplete data				
PS only	3	3	7	8
DW-MRI only	-	1	2	1
Complete data	229	206	188	164

Note. y = year, PS = Processing speed. DW-MRI = diffusion-weighted MRI.
