# Supplementary material for: Lagged Coupled Changes Between White Matter Microstructure and Processing Speed in Healthy Aging: A Longitudinal Investigation
Source: Front Aging Neurosci. 2019 Nov 21;11:298. doi: 10.3389/fnagi.2019.00298 (PMC6881240; doi:10.3389/fnagi.2019.00298)
Supplement: Supplementary file 4 [file Table_2.pdf]

Table S2 (Part 1)

Zero-order correlations between raw FA values (range 0 to 1) for 10 WM tracts within and across measurement occasions

		1	2	3	4	5	6	7	8	9	10	11	12	13	14	15	16	17	18	19	20
1	FMAJ$_{base}$	1.00																			
2	FMIN$_{base}$	0.64	1.00																		
3	SLF$_{base}$	0.60	0.67	1.00																	
4	ILF$_{base}$	0.57	0.62	0.69	1.00																
5	IFOF$_{base}$	0.60	0.70	0.74	0.77	1.00															
6	ATR$_{base}$	0.66	0.75	0.68	0.59	0.73	1														
7	UNC$_{base}$	0.43	0.56	0.49	0.54	0.62	0.55	1													
8	CCG$_{base}$	0.56	0.68	0.59	0.53	0.56	0.61	0.49	1												
9	CHC$_{base}$	0.32	0.26	0.39	0.52	0.36	0.33	0.31	0.34	1											
10	CST$_{base}$	0.14	0.19	0.40	0.41	0.39	0.21	0.32	0.20	0.27	1										
11	FMAJ$_{1y}$	**0.93**	0.62	0.55	0.52	0.54	0.6	0.40	0.50	0.29	*0.02*	1									
12	FMIN$_{1y}$	0.62	**0.93**	0.64	0.57	0.64	0.71	0.56	0.63	0.27	*0.11*	0.66	1								
13	SLF$_{1y}$	0.55	0.61	**0.90**	0.61	0.67	0.58	0.46	0.49	0.28	0.31	0.60	0.66	1							
14	ILF$_{1y}$	0.53	0.60	0.59	**0.86**	0.70	0.51	0.49	0.45	0.38	0.26	0.61	0.64	0.68	1						
15	IFOF$_{1y}$	0.54	0.64	0.68	0.67	**0.91**	0.64	0.58	0.51	0.29	0.29	0.60	0.71	0.76	0.78	1					
16	ATR$_{1y}$	0.58	0.67	0.65	0.57	0.67	**0.87**	0.49	0.54	0.34	*0.13*	0.63	0.74	0.67	0.61	0.75	1				
17	UNC$_{1y}$	0.39	0.54	0.51	0.54	0.65	0.51	**0.81**	0.44	0.26	0.23	0.46	0.62	0.60	0.66	0.73	0.62	1			
18	CCG$_{1y}$	0.53	0.67	0.57	0.52	0.57	0.60	0.49	**0.96**	0.31	0.16	0.56	0.68	0.56	0.53	0.60	0.63	0.52	1		
19	CHC$_{1y}$	0.31	0.33	0.33	0.47	0.34	0.27	0.22	0.30	**0.65**	*0.04*	0.41	0.38	0.39	0.57	0.42	0.39	0.38	0.38	1	
20	CST$_{1y}$	*0.03*	0.16	0.36	0.37	0.37	*0.13*	0.23	*0.11*	0.15	**0.75**	*0.10*	0.18	0.46	0.42	0.43	0.25	0.37	0.19	0.29	1
21	FMAJ$_{2y}$	0.95	0.58	0.58	0.49	0.56	0.65	0.43	0.53	0.24	*0.12*	**0.93**	0.59	0.59	0.52	0.57	0.61	0.42	0.51	0.26	*0.07*
22	FMIN$_{2y}$	0.57	0.93	0.64	0.55	0.62	0.74	0.57	0.63	0.22	0.18	0.56	**0.93**	0.65	0.59	0.64	0.71	0.60	0.64	0.32	0.20
23	SLF$_{2y}$	0.59	0.66	0.91	0.59	0.68	0.62	0.48	0.54	0.28	0.36	0.57	0.67	**0.95**	0.58	0.70	0.67	0.54	0.57	0.30	0.40
24	ILF$_{2y}$	0.58	0.60	0.61	0.87	0.71	0.53	0.53	0.48	0.42	0.31	0.58	0.62	0.65	**0.89**	0.73	0.57	0.59	0.53	0.47	0.35
25	IFOF$_{2y}$	0.59	0.66	0.70	0.67	0.90	0.68	0.59	0.56	0.29	0.34	0.57	0.67	0.73	0.69	**0.92**	0.69	0.67	0.59	0.33	0.38
26	ATR$_{2y}$	0.60	0.68	0.65	0.50	0.64	0.90	0.46	0.59	0.27	0.18	0.55	0.69	0.64	0.47	0.63	**0.91**	0.52	0.61	0.25	0.18
27	UNC$_{2y}$	0.48	0.56	0.54	0.49	0.61	0.54	0.80	0.50	0.27	0.22	0.48	0.61	0.56	0.53	0.65	0.57	**0.88**	0.53	0.31	0.25

28	CCG_{2y}	0.50	0.61	0.56	0.46	0.53	0.58	0.46	0.96	0.27	0.17	0.46	0.60	0.54	0.44	0.54	0.58	0.47	**0.97**	0.31	*0.15*
29	CHC_{2y}	0.30	0.38	0.38	0.48	0.38	0.29	0.28	0.42	0.70	*0.04*	0.32	0.41	0.37	0.47	0.40	0.38	0.38	0.44	**0.81**	0.18
30	CST_{2y}	*0.09*	0.18	0.38	0.31	0.36	0.16	0.27	0.16	*0.12*	0.77	*0.05*	0.17	0.41	0.29	0.38	0.21	0.32	0.20	*0.14*	**0.82**
31	$FMAJ_{4y}$	0.90	0.56	0.48	0.44	0.49	0.59	0.39	0.53	0.24	*0.05*	0.90	0.57	0.49	0.49	0.49	0.56	0.43	0.5	0.24	*0.05*
32	$FMIN_{4y}$	0.56	0.89	0.64	0.50	0.61	0.67	0.46	0.63	*0.15*	*0.11*	0.58	0.89	0.62	0.53	0.63	0.67	0.56	0.63	0.28	0.19
33	SLF_{4y}	0.50	0.61	0.87	0.54	0.63	0.55	0.40	0.49	0.23	0.28	0.53	0.63	0.91	0.55	0.66	0.61	0.49	0.53	0.28	0.40
34	ILF_{4y}	0.53	0.52	0.53	0.82	0.63	0.45	0.44	0.46	0.36	0.21	0.59	0.54	0.56	0.85	0.64	0.49	0.52	0.48	0.42	0.31
35	$IFOF_{4y}$	0.55	0.64	0.65	0.61	0.86	0.67	0.51	0.59	0.22	0.22	0.55	0.65	0.66	0.65	0.88	0.69	0.64	0.62	0.27	0.33
36	ATR_{4y}	0.59	0.64	0.63	0.48	0.64	0.84	0.43	0.60	0.26	*0.11*	0.56	0.68	0.59	0.47	0.64	0.89	0.54	0.63	0.23	0.17
37	UNC_{4y}	0.44	0.54	0.49	0.49	0.58	0.49	0.74	0.49	0.21	*0.14*	0.50	0.59	0.50	0.54	0.64	0.53	0.84	0.53	0.31	0.25
38	CCG_{4y}	0.56	0.67	0.59	0.51	0.58	0.61	0.45	0.95	0.28	*0.13*	0.53	0.67	0.55	0.48	0.59	0.62	0.48	0.96	0.32	0.17
39	CHC_{4y}	0.29	0.30	0.31	0.43	0.25	0.22	0.23	0.33	0.70	*0.02*	0.37	0.33	0.30	0.42	0.27	0.32	0.31	0.36	0.73	0.17
40	CST_{4y}	*0.07*	*0.15*	0.43	0.39	0.33	*0.11*	0.26	*0.13*	0.17	0.75	*0.06*	*0.15*	0.42	0.34	0.32	*0.14*	0.28	*0.16*	*0.14*	0.83
	M	0.55	0.36	0.36	0.40	0.45	0.36	0.39	0.40	0.42	0.54	0.55	0.37	0.36	0.40	0.45	0.36	0.39	0.40	0.41	0.54
	SD	0.03	0.02	0.02	0.02	0.02	0.02	0.02	0.04	0.03	0.02	0.03	0.02	0.02	0.02	0.02	0.02	0.02	0.04	0.03	0.02
	n	215	215	215	215	215	215	215	215	215	215	194	194	194	194	194	194	194	194	194	194

Note. FA values for bi-hemispheric tracts are averaged across hemispheres as described in the methods section. FMAJ = forceps major, FMIN = forceps minor, ILF = inferior longitudinal fasciculus, SLF = superior longitudinal fasciculus, IFOF = inferior fronto-occipital fasciculus, ATR = anterior thalamic radiation, UNC = uncinate fasciculus, CCG = cingulum cingulate gyrus, CHC = cingulum hippocampus, CST = corticospinal tract, base = baseline, y = year. Non-significant correlations ($p > 0.05$) are highlighted in italic font, correlations between the same tracts across measurement occasions are highlighted in bold font.

Table S2 (Part 2)

Zero-order correlations between raw FA values (range 0 to 1) for 10 WM tracts within and across measurement occasions

		21	22	23	24	25	26	27	28	29	30	31	32	33	34	35	36	37	38	39	40
1	FMAJ$_{base}$																				
2	FMIN$_{base}$																				
3	SLF$_{base}$																				
4	ILF$_{base}$																				
5	IFOF$_{base}$																				
6	ATR$_{base}$																				
7	UNC$_{base}$																				
8	CCG$_{base}$																				
9	CHC$_{base}$																				
10	CST$_{base}$																				
11	FMAJ$_{1y}$																				
12	FMIN$_{1y}$																				
13	SLF$_{1y}$																				
14	ILF$_{1y}$																				
15	IFOF$_{1y}$																				
16	ATR$_{1y}$																				
17	UNC$_{1y}$																				
18	CCG$_{1y}$																				
19	CHC$_{1y}$																				
20	CST$_{1y}$																				
21	FMAJ$_{2y}$	1																			
22	FMIN$_{2y}$	0.58	1																		
23	SLF$_{2y}$	0.62	0.68	1																	
24	ILF$_{2y}$	0.60	0.61	0.67	1																
25	IFOF$_{2y}$	0.64	0.70	0.77	0.77	1															
26	ATR$_{2y}$	0.65	0.75	0.70	0.56	0.74	1														
27	UNC$_{2y}$	0.53	0.64	0.61	0.64	0.72	0.60	1													
28	CCG$_{2y}$	0.54	0.65	0.60	0.52	0.60	0.65	0.56	1												
29	CHC$_{2y}$	0.32	0.41	0.39	0.52	0.40	0.35	0.41	0.45	1											
30	CST$_{2y}$	*0.14*	0.17	0.45	0.37	0.40	0.24	0.37	0.24	0.20	1										

31	FMAJ$_{4y}$	**0.90**	0.55	0.48	0.52	0.52	0.57	0.47	0.49	0.22	*0.04*	1									
32	FMIN$_{4y}$	0.56	**0.91**	0.64	0.54	0.65	0.70	0.57	0.60	0.34	*0.16*	0.62	1								
33	SLF$_{4y}$	0.52	0.64	**0.90**	0.60	0.69	0.62	0.55	0.52	0.28	0.37	0.58	0.70	1							
34	ILF$_{4y}$	0.53	0.51	0.52	**0.86**	0.63	0.45	0.51	0.45	0.40	0.23	0.61	0.59	0.65	1						
35	IFOF$_{4y}$	0.56	0.65	0.66	0.68	**0.89**	0.69	0.64	0.59	0.26	0.30	0.63	0.74	0.76	0.74	1					
36	ATR$_{4y}$	0.64	0.70	0.63	0.51	0.70	**0.92**	0.56	0.62	0.28	*0.17*	0.68	0.77	0.70	0.57	0.80	1				
37	UNC$_{4y}$	0.47	0.57	0.49	0.57	0.63	0.49	**0.84**	0.49	0.29	0.26	0.57	0.67	0.61	0.64	0.74	0.64	1			
38	CCG$_{4y}$	0.56	0.68	0.58	0.54	0.63	0.66	0.55	**0.96**	0.42	0.19	0.60	0.70	0.60	0.54	0.67	0.70	0.58	1		
39	CHC$_{4y}$	0.26	0.28	0.24	0.42	0.23	0.25	0.29	0.32	**0.79**	*0.07*	0.38	0.41	0.40	0.54	0.36	0.37	0.44	0.40	1	
40	CST$_{4y}$	*0.11*	*0.17*	0.42	0.37	0.35	*0.15*	0.32	*0.15*	*0.09*	**0.83**	*0.11*	*0.15*	0.45	0.37	0.35	0.21	0.32	0.18	0.2	1
	M	0.55	0.37	0.35	0.40	0.44	0.36	0.39	0.39	0.41	0.54	0.55	0.36	0.36	0.40	0.44	0.36	0.39	0.39	0.41	0.55
	SD	0.03	0.02	0.02	0.02	0.02	0.02	0.02	0.04	0.03	0.02	0.03	0.03	0.02	0.02	0.02	0.03	0.02	0.04	0.03	0.02
	n	173	173	173	173	173	173	173	173	173	173	153	153	153	153	153	153	153	153	153	153

Note. FA values for bi-hemispheric tracts are averaged across hemispheres as described in the methods section. FMAJ = forceps major, FMIN = forceps minor, ILF = inferior longitudinal fasciculus, SLF = superior longitudinal fasciculus, IFOF = inferior fronto-occipital fasciculus, ATR = anterior thalamic radiation, UNC = uncinate fasciculus, CCG = cingulum cingulate gyrus, CHC = cingulum hippocampus, CST = corticospinal tract, base = baseline, y = year. Non-significant correlations ($p > 0.05$) are highlighted in italic font, correlations between the same tracts across measurement occasions are highlighted in bold font.
