# Supplementary material for: Lagged Coupled Changes Between White Matter Microstructure and Processing Speed in Healthy Aging: A Longitudinal Investigation
Source: Front Aging Neurosci. 2019 Nov 21;11:298. doi: 10.3389/fnagi.2019.00298 (PMC6881240; doi:10.3389/fnagi.2019.00298)
Supplement: Supplementary file 5 [file Table_3.pdf]

Table S3

Pearson-correlations between raw processing speed values for four cognitive tasks within and across measurement occasions.

		1	2	3	4	5	6	7	8	9	10	11	12	13	14	15	16
1	DIGSY$_{\text{base}}$	1.00															
2	IPT1$_{\text{base}}$	0.53	1.00														
3	LPS14$_{\text{base}}$	0.69	0.57	1.00													
4	TMTA$_{\text{base}}$	0.47	0.48	0.50	1.00												
5	DIGSY$_{\text{1y}}$	**0.84**	0.56	0.66	0.49	1.00											
6	IPT$_{\text{1y}}$	0.58	**0.78**	0.59	0.50	0.58	1.00										
7	LPS14$_{\text{1y}}$	0.69	0.52	**0.77**	0.45	0.68	0.58	1.00									
8	TMTA$_{\text{1y}}$	0.49	0.48	0.45	**0.53**	0.54	0.50	0.44	1.00								
9	DIGSY$_{\text{2y}}$	0.80	0.53	0.65	0.46	**0.84**	0.53	0.67	0.53	1.00							
10	IPT$_{\text{2y}}$	0.58	0.79	0.61	0.53	0.54	**0.80**	0.59	0.50	0.55	1.00						
11	LPS14$_{\text{2y}}$	0.71	0.57	0.79	0.57	0.70	0.62	**0.77**	0.53	0.70	0.63	1.00					
12	TMTA$_{\text{2y}}$	0.43	0.50	0.40	0.58	0.44	0.45	0.37	**0.61**	0.47	0.48	0.48	1.00				
13	DIGSY$_{\text{4y}}$	0.79	0.48	0.61	0.52	0.80	0.55	0.63	0.48	**0.81**	0.53	0.70	0.42	1.00			
14	IPT$_{\text{4y}}$	0.54	0.75	0.60	0.52	0.56	0.77	0.55	0.48	0.54	**0.77**	0.66	0.49	0.58	1.00		
15	LPS14$_{\text{4y}}$	0.66	0.39	0.69	0.41	0.57	0.49	0.72	0.36	0.63	0.52	**0.73**	0.36	0.69	0.54	1.00	
16	TMTA$_{\text{4y}}$	0.43	0.39	0.44	0.52	0.42	0.48	0.43	0.46	0.39	0.41	0.39	**0.51**	0.43	0.49	0.45	1.00
	M	57.57	47.87	20.35	-37.83	60.12	49.95	20.78	-38.05	60.75	50.64	20.40	-36.95	60.67	48.72	19.58	-37.55
	SD	12.34	9.94	4.61	11.02	13.10	10.24	4.47	9.40	12.56	10.33	4.89	10.57	13.01	10.66	4.72	10.95
	n	231	231	231	225	208	208	209	207	192	192	194	191	168	167	168	163

Note. DIGSY = digit symbol test, LPS14 = Leistungsprüfsystem 14, IPT = identical pictures test, TMTA = trail making test part A, base = baseline, y = year. Values for all tasks are raw scores, coded such that higher values represent better performance. All correlations presented here are significant ($p < 0.001$). Correlations between the same tasks across measurement occasions are highlighted in bold font.
