# Supplementary material for: Lagged Coupled Changes Between White Matter Microstructure and Processing Speed in Healthy Aging: A Longitudinal Investigation
Source: Front Aging Neurosci. 2019 Nov 21;11:298. doi: 10.3389/fnagi.2019.00298 (PMC6881240; doi:10.3389/fnagi.2019.00298)
Supplement: Supplementary file 6 [file Table_4.pdf]

Table S4

Results from univariate LCS models of FA in 10 WM Tracts

Variable		Mean (μ)			Variance (σ^2)			Model fit				
		Estimate	*SE*	*p* value	Estimate	*SE*	*p* value	$\chi^2(df)$	χ^2/df	RMSEA [95% *CI*]	CFI	BIC
FMAJ	Intercept	55.536	0.668	**< .001**	8.818	0.946	**< .001**	37.851	1.46	0.044	0.990	4199.410
	Slope	-0.056	0.102	.585	0.028	0.016	.092	(26)		[0.000 - 0.073]		
FMIN	Intercept	37.956	0.501	**< .001**	4.277	0.361	**< .001**	45.397	1.75	0.057	0.983	3832.547
	Slope	-0.199	0.093	**.033**	0.038	0.014	**.006**	(26)		[0.027 - 0.084]		
SLF	Intercept	35.796	0.438	**< .001**	3.259	0.445	**< .001**	77.611	2.99	0.092	0.953	3595.481
	Slope	-0.219	0.077	**.004**	0.015	0.007	**.020**	(26)		[0.069 - 0.117]		
ILF	Intercept	40.033	0.438	**< .001**	3.176	0.341	**< .001**	35.914	1.38	0.041	0.988	3783.443
	Slope	-0.167	0.092	.069	0.019	0.011	.065	(26)		[0.000 - 0.070]		
IFOF	Intercept	44.501	0.517	**< .001**	4.320	0.468	**< .001**	34.428	1.32	0.037	0.992	3857.086
	Slope	-0.177	0.087	**.042**	0.022	0.011	.052	(26)		[0.000 - 0.068]		
ATR	Intercept	36.516	0.501	**< .001**	3.855	0.450	**< .001**	61.275	2.36	0.076	0.966	3871.739
	Slope	-0.417	0.092	**< .001**	0.035	0.013	**.006**	(26)		[0.052 - 0.101]		
UNC	Intercept	39.026	0.495	**< .001**	3.892	0.399	**< .001**	57.639	2.22	0.072	0.958	4125.155
	Slope	-0.179	0.122	.141	0.051	0.020	**.011**	(26)		[0.047 - 0.098]		
CCG	Intercept	41.053	0.731	**< .001**	10.603	0.992	**< .001**	40.148	1.54	0.048	0.990	4060.060
	Slope	-0.347	0.089	**< .001**	0.019	0.012	.098	(26)		[0.012 - 0.077]		
CHC	Intercept	41.559	0.685	**< .001**	6.433	0.973	**< .001**	74.585	2.76	0.087	0.912	4760.960
	Slope	-0.140	0.161	.382	0.000[a]	-	-	(27)		[0.064 - 0.111]		
CST	Intercept	53.976	0.484	**< .001**	3.904	0.421	**< .001**	37.310	1.38	0.041	0.984	4213.115
	Slope	-0.274	0.113	**.015**	0.000[a]	-	-	(27)		[0.000 - 0.070]		

Note. Parameter estimates are unstandardized, and adjusted for effects of age at baseline, education, and gender (on intercept and slope), and for head motion at each measurement occasion. FA values are raw scores, multiplied by 100. Significant results ($p < 0.05$) are highlighted in bold font.

[a] fixed to 0 due to the estimation of a negative slope variance.
