# Supplementary material for: Lagged Coupled Changes Between White Matter Microstructure and Processing Speed in Healthy Aging: A Longitudinal Investigation
Source: Front Aging Neurosci. 2019 Nov 21;11:298. doi: 10.3389/fnagi.2019.00298 (PMC6881240; doi:10.3389/fnagi.2019.00298)
Supplement: Supplementary file 7 [file Table_5.pdf]

Table S5

Effects of covariates on intercept and slope in univariate LCS models of FA in 10 WM tracts

		Age_{base}			Education			Gender		
Tract		Estimate	*SE*	*p* value	Estimate	*SE*	*p* value	Estimate	*SE*	*p* value
FMAJ	Intercept	-0.219	0.046	**<.001**	0.047	0.248	.851	-0.276	0.427	.518
	Slope	-0.022	0.006	**<.001**	0.053	0.030	.076	0.012	0.053	.824
FMIN	Intercept	-0.181	0.030	**<.001**	-0.107	0.175	.542	-0.592	0.299	**.048**
	Slope	-0.012	0.006	**.042**	0.009	0.029	.752	0.050	0.047	.291
SLF	Intercept	-0.131	0.030	**<.001**	-0.055	0.148	.709	0.391	0.262	.136
	Slope	-0.007	0.004	.124	0.016	0.022	.463	0.013	0.035	.710
ILF	Intercept	-0.075	0.027	**.005**	-0.170	0.146	.245	0.283	0.256	.269
	Slope	-0.007	0.005	.175	0.005	0.024	.827	-0.007	0.043	.869
IFOF	Intercept	-0.146	0.033	**<.001**	-0.282	0.178	.114	0.435	0.305	.154
	Slope	-0.015	0.005	**.003**	-0.011	0.026	.668	0.045	0.044	.308
ATR	Intercept	-0.163	0.029	**<.001**	-0.057	0.165	.729	0.348	0.288	.227
	Slope	-0.017	0.005	**.001**	-0.029	0.029	.324	0.151	0.047	**.001**
UNC	Intercept	-0.115	0.030	**<.001**	-0.179	0.176	.309	0.014	0.304	.963
	Slope	-0.014	0.007	**.043**	-0.017	0.037	.649	0.115	0.062	.062
CCG	Intercept	-0.233	0.043	**<.001**	0.076	0.278	.786	-0.342	0.456	.453
	Slope	-0.017	0.006	**.002**	-0.003	0.027	.923	0.045	0.043	.293
CHC	Intercept	-0.053	0.033	.110	0.347	0.244	.155	0.794	0.388	**.041**
	Slope	-0.017	0.009	.052	-0.047	0.046	.304	-0.101	0.081	.213
CST	Intercept	0.002	0.029	.952	-0.004	0.164	.982	0.538	0.287	.061
	Slope	-0.017	0.006	**.004**	-0.019	0.032	.550	0.233	0.057	**<.001**

Note. Age_{base} = age at baseline. Parameter estimates are unstandardized. Significant results ($p < 0.05$) are highlighted in bold font.
