# Supplementary material for: Lagged Coupled Changes Between White Matter Microstructure and Processing Speed in Healthy Aging: A Longitudinal Investigation
Source: Front Aging Neurosci. 2019 Nov 21;11:298. doi: 10.3389/fnagi.2019.00298 (PMC6881240; doi:10.3389/fnagi.2019.00298)
Supplement: Supplementary file 8 [file Table_6.pdf]

Table S6

Effects of head motion on FA at each measurement occasion in univariate LCS models for 10 WM tracts

	Head Motion$_{base}$			Head Motion$_{1year}$			Head Motion$_{2years}$			Head Motion$_{4years}$		
Tract	Estimate	*SE*	*p* value	Estimate	*SE*	*p* value	Estimate	*SE*	*p* value	Estimate	*SE*	*p* value
FMAJ	-1.133	0.679	.095	-0.900	0.547	.100	-0.923	0.532	.083	-1.656	0.859	.054
FMIN	-1.794	0.630	**.004**	-0.547	0.506	.280	-0.758	0.475	.111	-1.283	0.861	.136
SLF	-0.933	0.493	.058	-2.061	0.355	**<.001**	-1.775	0.350	**<.001**	-0.613	0.656	.350
ILF	-0.982	0.609	.107	-2.282	0.431	**<.001**	-1.914	0.413	**<.001**	-0.541	0.692	.435
IFOF	-1.421	0.622	**.022**	-1.383	0.420	**.001**	-1.710	0.415	**<.001**	-1.696	0.771	**.028**
ATR	-2.610	0.629	**<.001**	-1.812	0.465	**<.001**	-1.825	0.409	**<.001**	-1.763	0.752	**.019**
UNC	-0.958	0.880	.276	-1.104	0.602	.067	-0.959	0.561	.088	-0.649	1.008	.519
CCG	-1.588	0.636	**.013**	-1.562	0.461	**.001**	-1.931	0.509	**<.001**	-2.034	0.803	**.011**
CHC	-3.094	1.778	.082	-6.301	1.072	**<.001**	-4.810	0.945	**<.001**	-2.143	1.244	.085
CST	-2.596	0.879	**.003**	-2.229	0.622	**<.001**	-2.434	0.676	**<.001**	-1.081	0.941	.250

Note. Base = baseline. Parameter estimates are unstandardized. Significant results ($p < 0.05$) are highlighted in bold font.
