# Supplementary material for: Lagged Coupled Changes Between White Matter Microstructure and Processing Speed in Healthy Aging: A Longitudinal Investigation
Source: Front Aging Neurosci. 2019 Nov 21;11:298. doi: 10.3389/fnagi.2019.00298 (PMC6881240; doi:10.3389/fnagi.2019.00298)
Supplement: Supplementary file 9 [file Table_7.pdf]

Table S7

Results from the final univariate LCS model for processing speed with four indicators

	Estimate	*SE*	*p* value
Factor Loadings			
IPT	1[a]	-	-
DIGSY	1.419	0.112	**<.001**
LPS14	0.507	0.035	**<.001**
TMTA	0.831	0.065	**<.001**
Intercept			
Mean (μ)	48.459	1.453	**<.001**
Variance (σ^2)	40.430	6.042	**<.001**
Slope			
Mean (μ)	-0.567	0.280	**.043**
Variance (σ^2)	0.441	0.161	**.006**
Retest			
Mean (μ)	2.032	0.343	**<.001**
Variance (σ^2)	0.000 [b]	-	-
Covariates			
Age_{base} → intercept	-0.645	0.096	**<.001**
Age_{base} → slope	-0.049	0.017	**.004**
Educ → intercept	1.246	0.554	**.025**
Educ → slope	0.069	0.089	.436
Gender → intercept	0.164	0.926	.859
Gender → slope	-0.025	0.163	.879

Note. Educ = education. Estimates are unstandardized. Factor loadings of the manifest indicators on the latent factor and intercepts of the manifest indicators (not shown in this table) are fixed to equality across measurement occasions (i.e., strong measurement invariance). Significant results ($p < 0.05$) are highlighted in bold font.

[a] this factor loading was fixed to 1 to set the scale of the latent processing speed factor.

[b] fixed to 0 due to the estimation of a negative slope variance.
